# On‐Demand Deconstructable Thermosets Through Cleavable Comonomer and Thermolatent Base Synergy

**DOI:** 10.1002/anie.6855232

**Published:** 2026-08-06

**Authors:** Sophia Kouider, Loic Buchon, Luna Choulot, Paul Greuet, Julie Bratasanu, Patrick Désirée, Jessica Mauriello, Elsa Maarek, Jean‐Louis Clément, Laurence Charles, Emmanuelle Gastaldi, Damien Montarnal, Olivier Soppera, Didier Gigmes, Catherine Lefay, Yohann Guillaneuf

**Affiliations:** ^1^ Aix‐Marseille Université, CNRS, Institut De Chimie Radicalaire, UMR 7273 Marseille France; ^2^ Université De Haute‐Alsace CNRS Mulhouse France; ^3^ Université De Strasbourg Strasbourg France; ^4^ UMR IATE L'Institut Agro Montpellier Université De Montpellier, INRAE Montpellier France; ^5^ CPE Lyon CNRS Catalysis Polymerization Processes and Materials Univ. Lyon Lyon France

**Keywords:** degradable thermoset, on‐demand deconstructable thermosets, radical ring‐opening polymerization, ring‐opening metathesis polymerization, thermolatent base/acid

## Abstract

Thermosets provide superior chemical and mechanical properties critical for high‐performance applications, but their permanent crosslinked networks severely limit recyclability and end‐of‐life (EOL) management. Traditional approaches relying on cleavable comonomers enable degradation only through slow diffusion of acidic or basic solutions, hindering practical implementation. Here, on‐demand deconstructable thermosets are developed by incorporating stimuli‐responsive thermolatent bases alongside cleavable comonomers prior to polymerization. These latent species remain inert during thermal or photoinitiated curing, including vat photopolymerization 3D printing, preserving classical thermoset performance. Upon near‐infrared photothermal or thermal activation (>100°C), rapid network deconstruction yields soluble branched oligomers, enabling efficient chemical recycling without aggressive solvents during service life. This strategy is demonstrated using thermolatent 1,5,7‐triazabicyclo[4.4.0]dec‐5‐ene derivatives with radical copolymerization of dibenzo[*c*,*e*]oxepine‐5‐thione with styrene/acrylic monomers, as well as ring‐opening metathesis polymerization of dicyclopentadiene with silyl ether‐containing cyclic olefins. The approach offers programmable degradation for sustainable high‐performance thermosets.

## Graphical abstract

Thermosets, valued for their chemical and mechanical properties, resist recycling due to their cross‐linked structure. Weak bonds via comonomers enable degradability, but diffusion limits effectiveness. We incorporate thermolatent bases before polymerization (thermal or 3D‐VAT printing). NIR or thermal activation (>100°C) triggers depolymerization, making networks soluble. This is demonstrated with DOT‐acrylate radical polymerization and dicyclopentadiene/silyl ether ROMP with thermolatent TBD compounds.

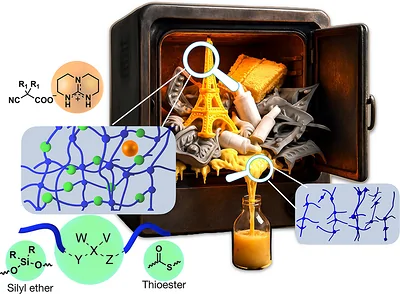

## 1 Introduction

Images of overflowing landfills and inappropriate garbage dumping into rivers and seas have made the macro‐scale environmental contamination caused by plastics in many forms apparent throughout the past few decades [1, 2, 3]. Common thermoplastics offer a range of recycling possibilities, from mechanical recycling to chemical recycling, the latter encompassing either degradation into low‐molar‐mass fragments or selective depolymerization to recover the original monomers, thereby enabling closed‐loop recycling. In contrast, thermosets, with their crosslinked networks, deliver superior mechanical strength, chemical resistance, and thermal stability compared to linear thermoplastics, making them indispensable in demanding applications ranging from aerospace composites to 3D‐printed structural components. Their natural cross‐linked structure, however, prevents any recycling, reprocessing, or degradability, contributing significantly to long‐term environmental burdens [4, 5, 6]. To address these challenges, dynamic covalent chemistries such as vitrimers have enabled reprocessing through bond exchange at elevated temperatures [7, 8, 9, 10], while multifunctional monomers with central degradable cores facilitate network disassembly [11]. However, both approaches generally require a complete redesign of the network by incorporating either dynamic linkages or new multifunctional monomers bearing degradable cores, which often leads to modifications in the thermomechanical properties compared to conventional thermosets. A particularly versatile strategy involves the comonomer approach, whereby low loadings (5–10 mol%) of monofunctional additives introduce cleavable bonds selectively into polymer backbones rather than crosslink junctions (Figure 1a). This concept was theorized by Johnson et al. [12] who demonstrated theoretically that 5–10 mol% of such an additive suffices to enable thermoset deconstruction. This method thus preserves native thermoset properties while enabling chemical degradation into soluble oligomers, and has been successfully applied across radical polymerization [13, 14, 15], ring‐opening metathesis polymerization (ROMP) [12, 16, 17, 18, 19, 20], and additive manufacturing techniques [21, 22].

**FIGURE 1:**
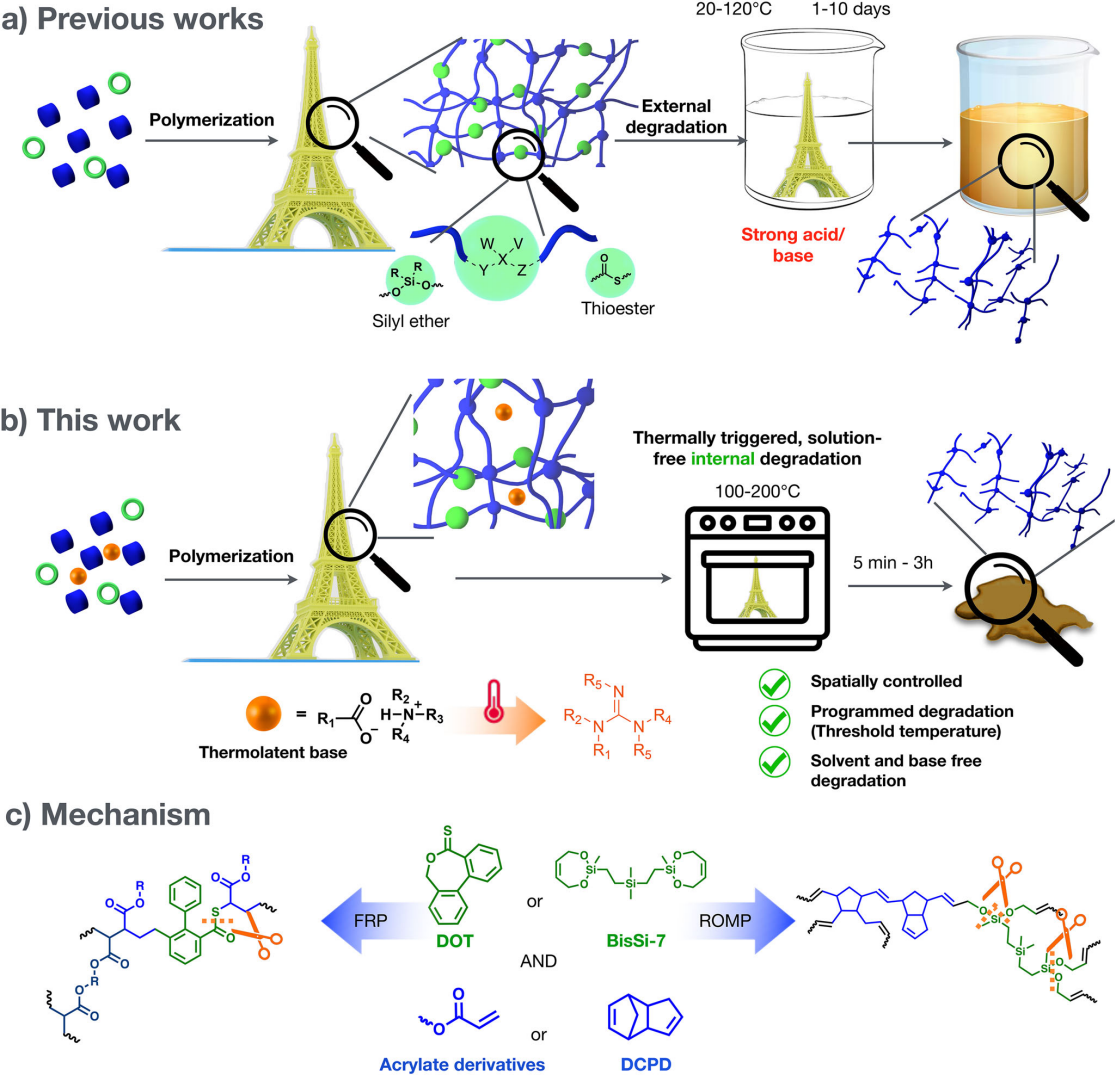
(a) The comonomer approach to prepare degradable/recyclable thermoset. The labile bond is located into the strands and not into the knots. (b) Concept of the self‐destruct thermosets combining the comonomer approach and thermolatent activation of the degrading species. Once activated the materials turned a dark brown color. (c) Mechanism of the self‐destruct thermosets using radical ring‐opening polymerization or ring‐opening metathesis polymerization.

The comonomer approach, which relies on introducing labile/cleavable bonds onto the polymer backbone to combine the material properties of the conventional materials with the possibility to degrade them via a chemical treatment, is becoming increasingly popular whatever the polymerization technique and has been recently reviewed [23]. Despite these advances, backbone‐cleavable thermosets typically rely on external triggers, acidic or basic solutions, that require slow diffusion into the network, often necessitating harsh conditions (e.g., elevated temperatures, concentrated reagents, or prolonged exposure) for complete deconstruction [23]. For example, in the case of HDPE mimics containing few ester moieties, the objects were treated at 120°C in EtOH (at 80 g polymer per liter) containing 10 wt% KOH for 24 h to obtain a complete degradation [24]. Similarly, Guillaneuf and coworkers [21] obtained faster degradation depending on the experimental degradation conditions in the basic degradation of 3D printed cross‐linked objects. The use of external degradation conditions is also a limitation to the development of this approach, since it requires that the degradation is performed by operators qualified to handle these chemicals in secured facilities, limiting practical end‐of‐life (EOL) management by end‐users. Here, on‐demand deconstructable thermosets with built‐in programmable degradation are realized by integrating thermolatent bases with cleavable comonomers directly into the network prior to curing (Figure 1b). These latent species remain inert during thermal or photoinitiated polymerization, ensuring compatibility with established processing methods, including vat photopolymerization. Upon thermal (>100°C) or near‐infrared photothermal activation, in situ generation of a strong organic base triggers rapid backbone cleavage, yielding soluble branched oligomers without requiring external aggressive solvents during service life.

This internal activation strategy is demonstrated across diverse systems: radical copolymerization of dibenzo[*c*,*e*]oxepine‐5‐thione (DOT) with styrene or acrylic monomers, and ROMP of dicyclopentadiene (DCPD) with silyl ether‐containing cyclic olefins, both incorporating tailored thermolatent 1,5,7‐triazabicyclo[4.4.0]dec‐5‐ene (TBD) derivatives (Figure 1c). The approach proves highly versatile, encompassing bulk thermal polymerization and vat photopolymerization 3D printing, as well as pure thermal (>100°C) and near‐infrared photothermal triggering—the latter enabling spatially controlled deconstruction. Practical utility is illustrated by the on‐demand thermal release of an embedded 3D‐printed object from a thermoset matrix and the development of an all‐organic thermal fuse exhibiting precise, temperature‐dependent programmable failure. Building upon prior proof‐of‐concept with photolatent bases in solution‐phase systems [13], this platform establishes stimuli‐responsive, self‐degrading high‐performance thermosets compatible with established manufacturing techniques, thereby advancing truly circular polymeric materials.

## 2 Results and Discussions

### 2.1 Thermally Triggered Vinyl‐Based Network Degradation

Among backbone‐cleavable chemistries accessible via the comonomer approach, radical copolymerization of vinyl monomers with thionolactones, particularly dibenzo[*c*,*e*]oxepine‐5‐thione (DOT), stands out for its compatibility with diverse processing techniques and established base‐mediated degradability. This system is selected as the primary platform to demonstrate thermally triggered internal deconstruction through integration of thermolatent bases.

Polyacrylate networks incorporating backbone thioester moieties are thus prepared via radical copolymerization of *n*‐butyl acrylate, tricyclo[5.2.1.02,6]decanedimethanol diacrylate (TcddA) as crosslinker, and dibenzo[*c*,*e*]oxepine‐5‐thione (DOT) as comonomer (4 equiv for 101 equiv of monomers, i.e., 3.8 mol% feed ratio), and anisole as solvent, following adapted literature protocols (Table S1, network B) [15, 25]. This amount of DOT was reported by Roth et al. [15, 25] to induce complete degradation of the cross‐linked network. Reference networks lacking DOT or thermolatent species are synthesized similarly. Additional networks incorporate 1–5 mol% of the thermolatent base 1,5,7‐triazabicyclo[4.4.0]dec‐5‐enyl cyanoacetate (ThermoTBD1), a quaternary ammonium salt reported by Schlögl and coworkers for clean thermal decarboxylation to release 1,5,7‐triazabicyclo[4.4.0]dec‐5‐ene (TBD) (Figure 2a and Table S1 network D and O‐R) [26].

**FIGURE 2:**
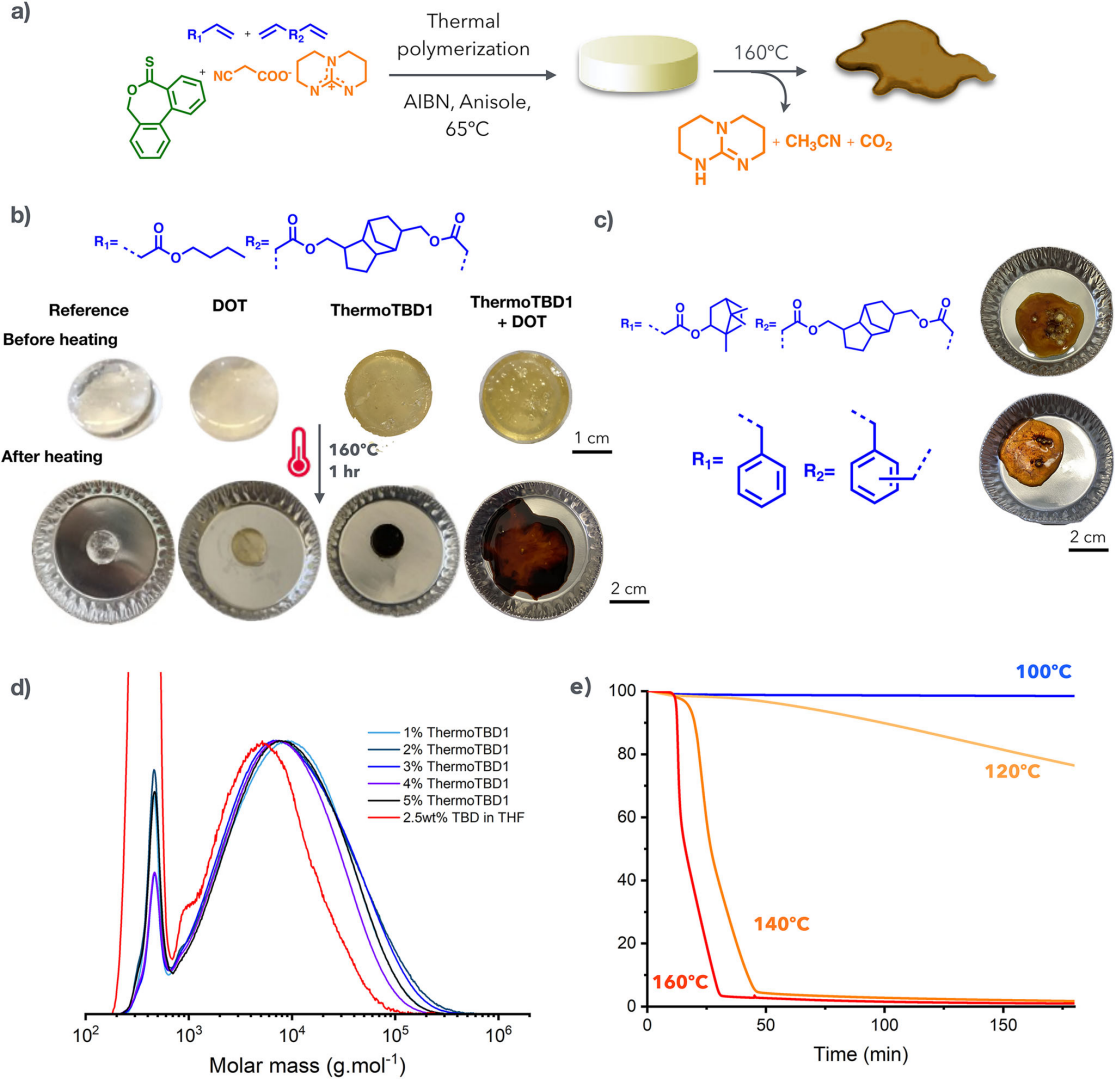
(a) Self‐destruct thermosets using thermally initiated radical ring‐opening copolymerization of DOT and vinyl monomers. (b) Acrylate network containing 3.8 mol% DOT and/or thermolatent ThermoTBD1 (0.25–1.25 equiv relative to DOT) before and after heating at 160°C (Table S1: Network D and O‐R). (c) Network of different vinyl monomers containing DOT and thermolatent ThermoTBD1 before and after heating at 160°C (Table S1: Network H and L). (d) SEC analyses of the degraded product. A DOT only containing network was degraded using a solution of TBD in THF (2.5 wt%) was also analyzed by SEC to compare the internal and external degradation. (e) TGA isothermal analyses of ThermoTBD1 between 80°C and 160°C. Polyacrylate networks containing DOT and thermolatent ThermoTBD1 before and after heating between 100°C and 140°C (Table S1: Network D).

Disk specimens (≈1 g) punched from these networks are subjected to thermal treatment. According to the work of Schlögl and coworkers [26], temperatures between 125°C and 160°C could lead to the in situ clean generation of TBD via the decarboxylation of the cyanoacetic acid salt. At 160°C, solvent evaporation occurred but reference polyacrylate network and networks containing only thioesters or only ThermoTBD1 retain structural integrity, exhibiting minor mass loss from volatiles and, in the latter case, coloration attributed to base release. In contrast, networks combining DOT‐derived thioesters and ThermoTBD1 undergo rapid deconstruction into dark viscous liquids (Figure 2b), with higher ThermoTBD1 (0.25–1.25 equiv relative to DOT) loadings accelerating the process (Figure S1). Size‐exclusion chromatography (SEC) of the recovered oligomers reveals consistent molecular weights (*M*_n_ = 4000–6000 g mol^−1^, *Đ* = 2.5–3.5) independent of ThermoTBD1 content, matching those obtained from conventional external base‐mediated degradation (Figures 2d and S1). MALDI‐TOF mass spectrometry further confirms mechanistic similarity since some distributions exhibit chain‐end structures analogous to those of linear polymers degraded in solution by TBD, while some other distributions with higher molecular weight contain multiple diacrylate junctions and higher chain‐end densities (Figure S2). Exact assignment of these species lies beyond the scope of this work; nevertheless, the analyses validate successful thermolatent activation to yield active TBD that behaves identically to an exogenous base in solution. Thermogravimetric analysis (TGA) of the pure thermolatent base indicates ThermoTBD1 activation at ≈156°C, consistent with literature (Figure 2e) [26]. Isothermal experiments of the pure thermolatent base establish inertness below 100°C (that is in good agreement with no degradation of the network over 3 h) and efficient decomposition above 140°C (in good agreement with the deconstruction), with intermediate behavior at 120°C (Figure 2e). This threshold enables selective on‐demand activation without compromising ambient stability. The approach proves robust across diverse vinyl networks, including high‐*T*_g_ systems based on isobornyl acrylate (*T*_g_ = 94°C) or styrene (*T*_g_ = 110°C) with comparable DOT incorporation (≈3.8 mol%), as well as acrylamide derivatives (Figures 2c and S3), all achieving complete latent deconstruction at 160°C.

To tune activation temperatures, alternative thermolatent bases are explored. Although thermolatent organic bases have been reported, no prior studies have systematically investigated structure–reactivity relationships to modulate their thermal decarboxylation thresholds. Here, deliberate structural modifications enable precise control: the dimethylcyanoacetate TBD analog (ThermoTBD2) displays enhanced thermal stability (*T*_d5%_ = 183°C), whereas the 1,1,3,3‐tetramethylguanidine cyanoacetate salt (ThermoTMG1) activates at markedly lower temperature (119°C) (Figure 3a,b). Importantly, all variants yield analogous degradation products regardless of activation temperature (Figure S4). Visual demonstration with dye‐loaded networks underscores this programmable behavior: ThermoTBD1‐containing samples deconstruct at 130°C, while ThermoTBD2 analogs remain intact until 170°C (Figure 3c). In all networks containing different thermolatent bases, SEC analyses of the degradation products were similar (Table S2 and Figure S4 for details), underlining that the degradation profile is solely related to the DOT incorporation into the network and not to the nature of the thermolatent base.

**FIGURE 3:**
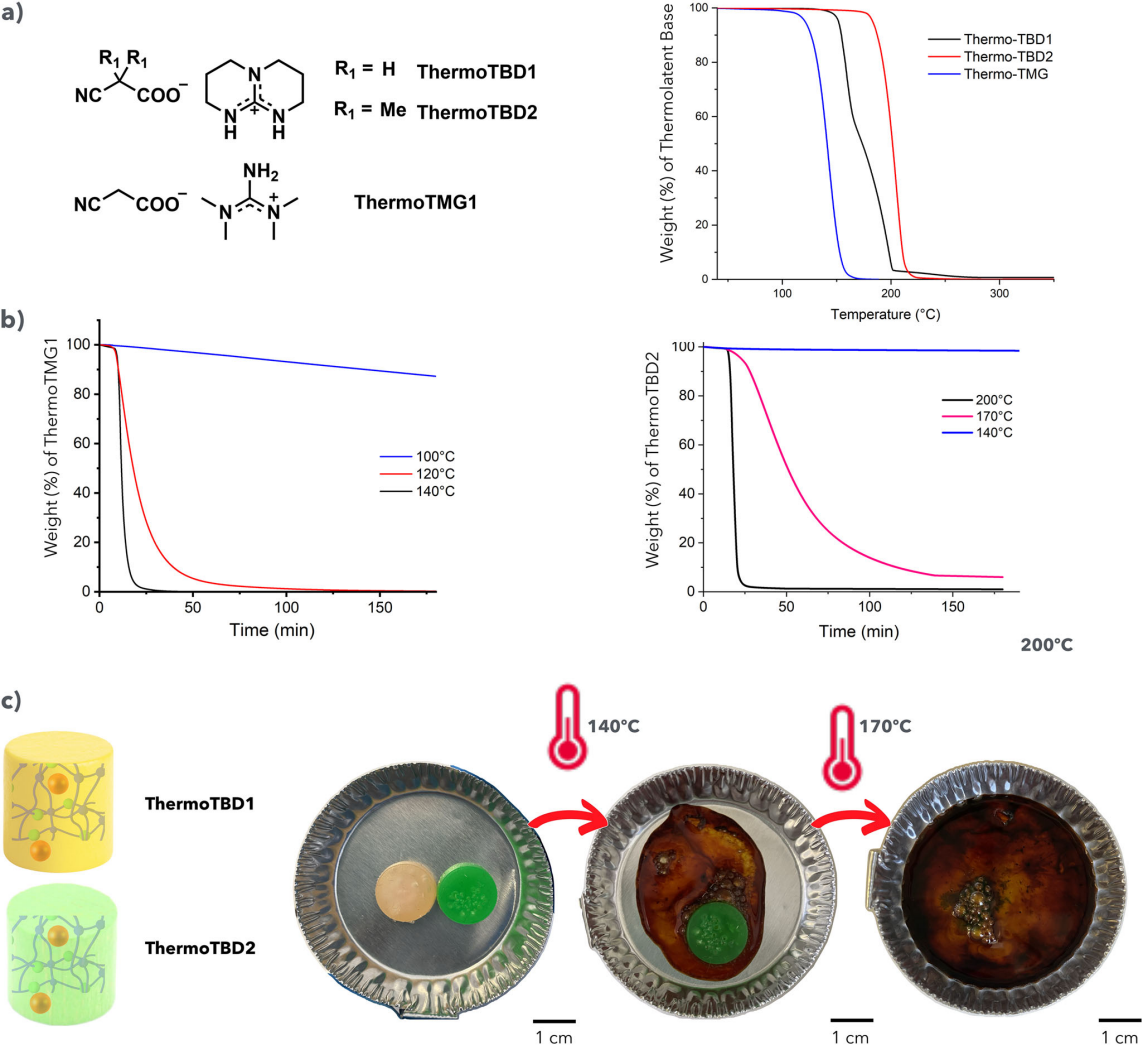
(a) Structure of the various thermolatent bases and TGA analyses of their decomposition. (b) Isotherms decomposition of the various thermolatent bases and photo of the degraded network. (c) Selective degradation of DOT‐polyacrylate networks (Table S1: Network D and S) containing ThermoTBD1 (yellow network) and ThermoTBD2 (green network).

Quantitative determination of the network degradation incorporating ThermoTBD2 latent base was assessed by dynamic rheology (1 Hz, 1% strain) while applying a temperature ramp (3 K min^−1^) from ambient to 180°C (Figure 4a). While all reference samples containing no DOT, no ThermoTBD2 or neither DOT nor ThermoTBD2 are well crosslinked and display stable properties in the 30°C–180°C range with moduli about 70–100 kPa, the sample containing both DOT and ThermoTBD2 shows a sudden decrease of moduli above 150°C. Dynamic rheology carried out at 30°C before and after the temperature ramp (Figure 4b—red) confirms the transition from a crosslinked network (*G*’ displaying a plateau about 70 kPa at low frequencies) to a low viscosity, unentangled liquid (*η*_0_ about 10 Pa s). For comparison the sample containing no DOT did not show significant changes of properties after the same treatment.

**FIGURE 4:**
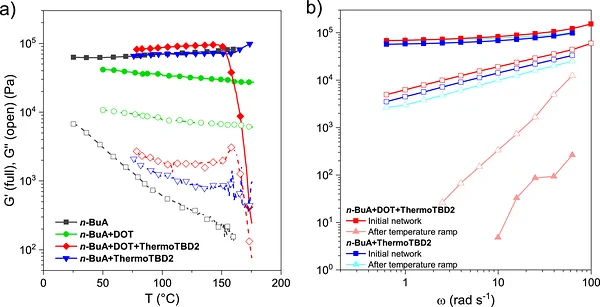
(a) Dynamic mechanical analysis (1% strain, 1 Hz, 3K min^−1^) of *n*‐BuA networks of various compositions (Table S1: Networks A, B, S, and T, respectively). (b) Dynamic rheology (1% strain) at 30°C before and after the temperature ramp.

### 2.2 ROMP‐Based Networks

To establish the generality of the internal activation strategy beyond radical polymerization, ring‐opening metathesis polymerization (ROMP) is investigated. Among reported cleavable comonomers for ROMP‐based thermosets [23, 27], silyl ether derivatives are selected, with initial focus on the seven‐membered cyclic monomer iPrSi‐7 (Figure 5a) [12, 16, 19, 20], readily synthesized from commercially available *cis*‐2‐butene‐1,4‐diol and dichloro‐diisopropylsilane and efficient for deconstructing dicyclopentadiene (DCPD) networks when at least 5–10 mol% was introduced [19].

**FIGURE 5:**
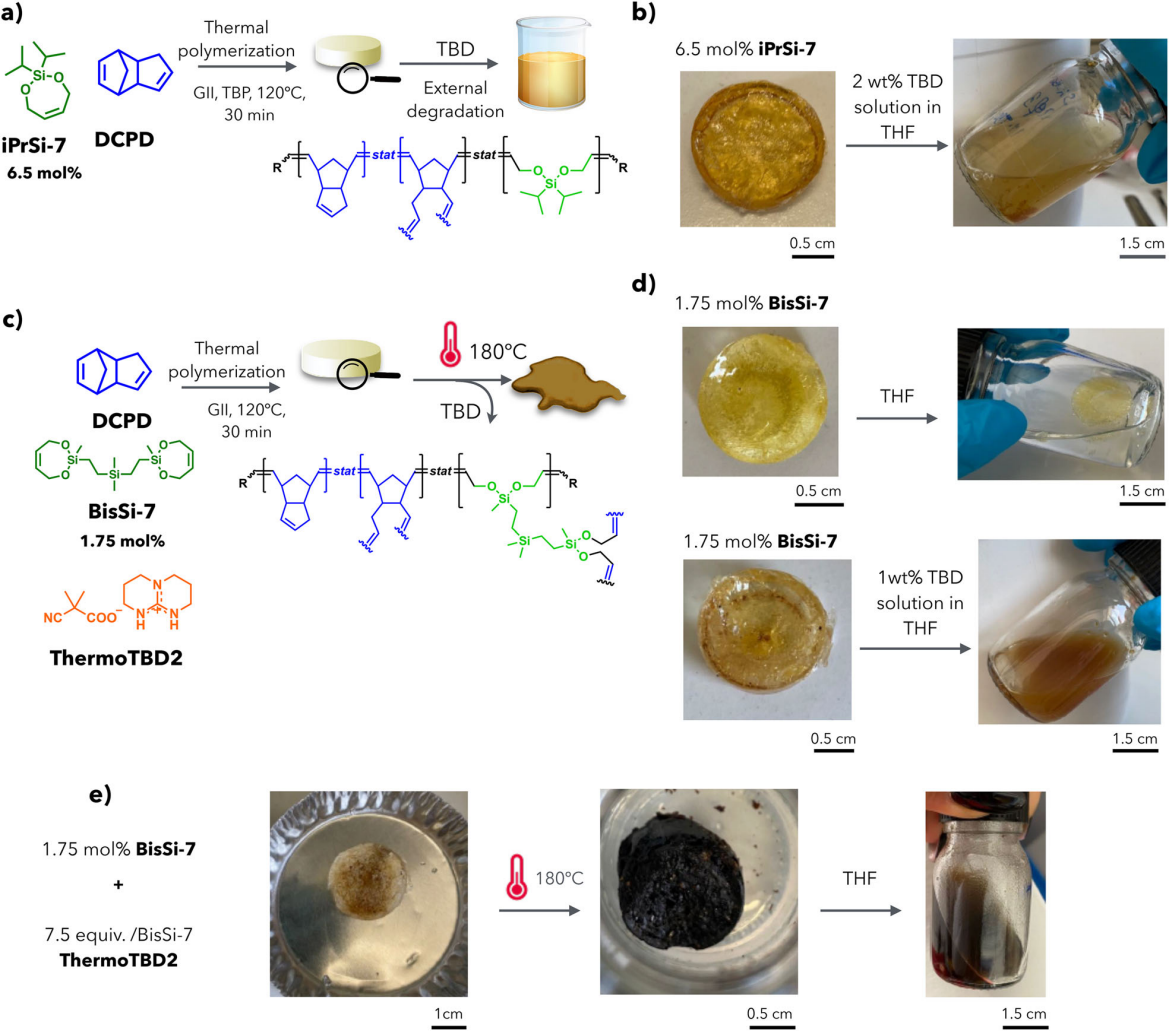
(a) Preparation of thermosets by ROMP of DCPD and iPrSi‐7 (6.5 mol%) using GII catalyst. (b) External degradation of the thermoset was then immersed in a 2 wt% TBD solution for 18 h. (c) Preparation of thermosets by ROMP of DCPD and BisSi‐7 (1.75 mol%) using GII catalyst. (d) External degradation of the thermoset was then immersed in a 1 wt% TBD solution for 18 h. (e) Internal degradation of the thermoset after heating at 180°C and immersed in THF at RT for 12 h.

Dicyclopentadiene (DCPD) networks incorporating 6.5 mol% iPrSi‐7 are then prepared according to literature protocols [18]. External degradation with TBD in THF (10 mL, 2 wt%, 3 equiv relative to iPrSi‐7, 12 h, room temperature) fully solubilizes these networks (Table S3), confirming base sensitivity, while reference networks lacking iPrSi‐7 remain intact (Figure 5b).

Networks co‐incorporating 1.6 equiv relative to iPrSi‐7 of ThermoTBD2 are then subjected to thermal activation (160°C, 1 h) followed by immersion in neat THF. ThermoTBD2 was chosen to limit thermal activation due to the overheating occurring during the curing of the resin. Although swelling and partial damage occur, complete dissolution is not achieved, attributed to insufficient in situ base generation relative to network density and limited ThermoTBD2 solubility in the resin.

Based on the external degradation solution, at least 3 equiv of ThermoTBD2 should have been added to ensure dissolution. It is difficult to consider adding more thermolatent base since the limit of this compound's solubility in the resin is almost reached.

To overcome this limitation, the recently reported strand‐fusing crosslinker bisSi‐7 by Johnson et al. [18] is adopted, enabling efficient degradation at lower loadings (≈4 mol%) (Figure 5c). Experimentally, corresponding DCPD networks with 1.75 mol% bisSi‐7 required only half of the external TBD amount for complete solubilization compared to iPrSi‐7 analogs (Figure 5d). Incorporation of 7.5 equiv of ThermoTBD2 relative to bisSi‐7 into these optimized networks (1.75 mol% bisSi‐7), followed by heating at 200°C and subsequent THF immersion (12 h, room temperature), results in full dissolution (Figures 5e, S5, and Table S3). This demonstrates the successful extension of thermolatent base‐triggered internal depolymerization to ROMP thermosets, further underscoring the platform's broad applicability across distinct polymerization chemistries. The characterization of the chain end groups and the mechanism of degradation will be detailed later in the end‐of‐life section.

### 2.3 Thermally Triggered Vinyl‐Based 3D‐Object Degradation

Having validated thermolatent base‐triggered internal deconstruction across FRP and ROMP‐derived thermosets, the approach is then extended to another polymerization process that is the vat photopolymerization 3D printing, a key additive manufacturing technique using multifunctional acrylate resins. In a previous study [21], we already demonstrated that thionolactone could be used as additive (2 wt%) to confer degradability to 3D objects.

Initial experiments employ pentaerythritol triacrylate (PETIA) as a model trifunctional resin to probe the approach in highly crosslinked photopolymerized networks typical of vat photopolymerization. Hollow cubic objects printed from resins containing 2 and 3 wt% DOT exhibit degradability upon external base treatment, consistent with prior experiments (Figure 6a) [21]. Specifically, immersion in 5 wt% KOH in THF/EtOH (1:1 v/v) achieves progressive solubilization, with degradation rates accelerating under elevated KOH concentrations or temperatures. In contrast, treatment with bulkier organic bases, 2.5 wt% TBD or *n*‐butylamine in THF, fails to induce deconstruction even after prolonged exposure (12–24 h), despite identical solvent systems (Figure 6a).

**FIGURE 6:**
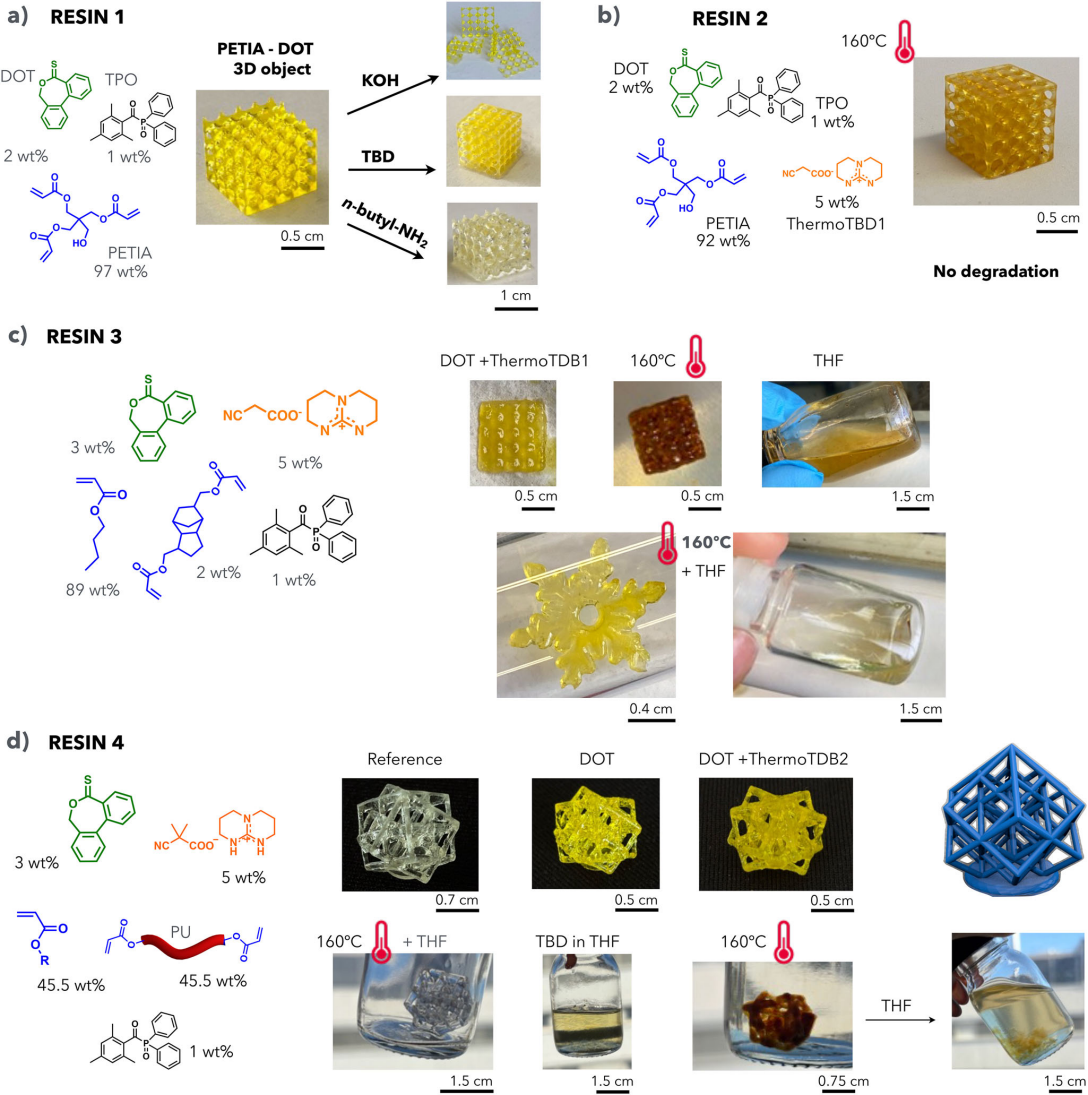
(a) Preparation of a 3D object via the VAT photopolymerization of a resin comprising PETIA, DOT and TPO as photoinitiator (Resin 1 in the SI). External degradation in solution using different bases (KOH, TBD, *n*‐butylamine). (b) Preparation of a 3D object using the same resin as in (a) but with ThermoTBD1 (5 wt %). After heating, the object just turned brown. (c) Preparation of a 3D object via the VAT photopolymerization of a resin comprising *n*BA, TcddA, DOT, ThermoTBD1, and TPO as photoinitiator (Resin 3 in the SI). Degradation by heating in an oven for 4 h followed by immersion in THF at RT. (d) Preparation of a prism object via the VAT photopolymerization of a resin comprising 50 wt% of a monoacrylate and 50 wt% of a PU‐diacrylate, DOT, ThermoTBD2, and TPO as photoinitiator. Degradation by heating in an oven for 4 h followed by immersion in THF at RT.

This marked selectivity highlights diffusion limitations in these densely crosslinked matrices. Networks derived from tri‐ and tetrafunctional acrylates exhibit negligible swelling across common solvents, severely restricting penetration of sterically demanding species such as TBD. Smaller hydroxide ions, however, could access backbone thioester sites more readily. These observations align with molecular dynamics simulations [27] revealing heterogeneous network topologies in photopolymerized multifunctional acrylates, characterized by compact, high‐density microgels that should impede transport of large molecules.

Co‐incorporation of 3 wt% of DOT and 5 wt% ThermoTBD1 (≈1.7 equiv relative to DOT) preserves printability without compromising resolution. Thermal activation at 160°C for 3 h induces characteristic coloration indicative of TBD release. However, subsequent THF immersion yields no dissolution (Figure 6b), likely due to persistent diffusion limitations in this densely crosslinked matrix.

To mitigate these constraints, lower‐crosslink‐density formulations are explored using a photoinitiator (TPO) and compositions analogous to thermally cured systems: n‐butyl acrylate with TcddA crosslinker (2 wt%) and DOT (3 wt%), in combination with 5 wt% of ThermoTBD1. Printed objects exhibit reduced resolution owing to moderate crosslinking but, upon heating, only samples containing both DOT and ThermoTBD1 fully dissolve in THF, while references remain intact (Figure 6c). This confirms the successful translation of internal deconstruction to photopolymerized networks.

In 3D photo‐printing, most of the resins are composed of a blend of reactive oligomers/polymers with a reactive diluent. Acrylate/methacrylate end‐capped oligo or polyurethane are one of the 3D‐printable main reactive oligomer/polymer resin components due to the toughness brought by the polyurethane segment. By using a model resin consisting in 50 wt% of a diacrylate polyurethane compound and 50 wt% of an monoacrylate as a reactive diluent, it is possible to prepare a resin that has a good printability and a low to moderate cross‐linking. Addition of 2 wt% DOT and 3 wt% ThermoTBD2 (chosen for superior solubility; ≈1.4 equiv) slightly slows photopolymerization kinetics, necessitating longer exposure times, yet yields high‐resolution prismatic objects (Figure 6d). Thermal treatment at 200°C triggers coloration and complete THF dissolution exclusively in dual‐additive samples, demonstrating that programmable self‐deconstruction can be seamlessly integrated with excellent printability in practical 3D printing formulations.

### 2.4 Spatially‐Triggered Degradation via IR Photothermal Activation

While thermal activation provides straightforward, shape‐independent degradation, spatially controlled dissociation remains highly desirable for applications requiring precise material removal or patterning. Previous work from our group demonstrated UV‐triggered degradation of polystyrene chains using photolatent bases, but this was limited to solution‐phase systems owing to the shallow penetration depth of low‐to‐moderate wavelength UV light [13]. Near‐infrared (NIR) photothermal heating offers an attractive alternative, as NIR radiation (700–1100 nm) penetrates deeper into polymers and can be focused to micrometer‐scale spots. NIR dyes convert absorbed light into localized heat, a principle already exploited in laser welding of polymers [28], achieving surface temperatures up to 300°C in thin films [29]. Recent studies have highlighted the potential of NIR‐induced heating for reprocessing, recycling [30], or sterilizing organic materials [31]. Notably, the commercially available dye IR813 *p*‐toluenesulfonate has proven highly efficient, generating temperatures exceeding 150°C in bulk samples containing only 0.1 wt% dye under 785 nm irradiation at >2 W cm^−^^2^.

To implement photothermal triggering, *n*‐butyl acrylate‐based networks incorporating 3.8 mol% DOT, 2.85 mol% ThermoTBD1, and 0.095 mol% IR813 are prepared according to established formulations (Figure 7a, Tables S1 – network D, and S4). Samples are exposed to an 808 nm laser at 20 W cm^−^^2^ for 6 min (Figure 7b). Reference networks without IR813 exhibit no heating and remain intact. Networks containing IR813 alone or combined with either DOT or ThermoTBD1 reach 150°C–160°C rapidly but retain structural integrity. Only samples combining all three components (DOT, ThermoTBD1, and IR813) undergo rapid melting and liquefaction, confirming synergistic activation of the thermolatent base and subsequent thioester cleavage (Figure 7c).

**FIGURE 7:**
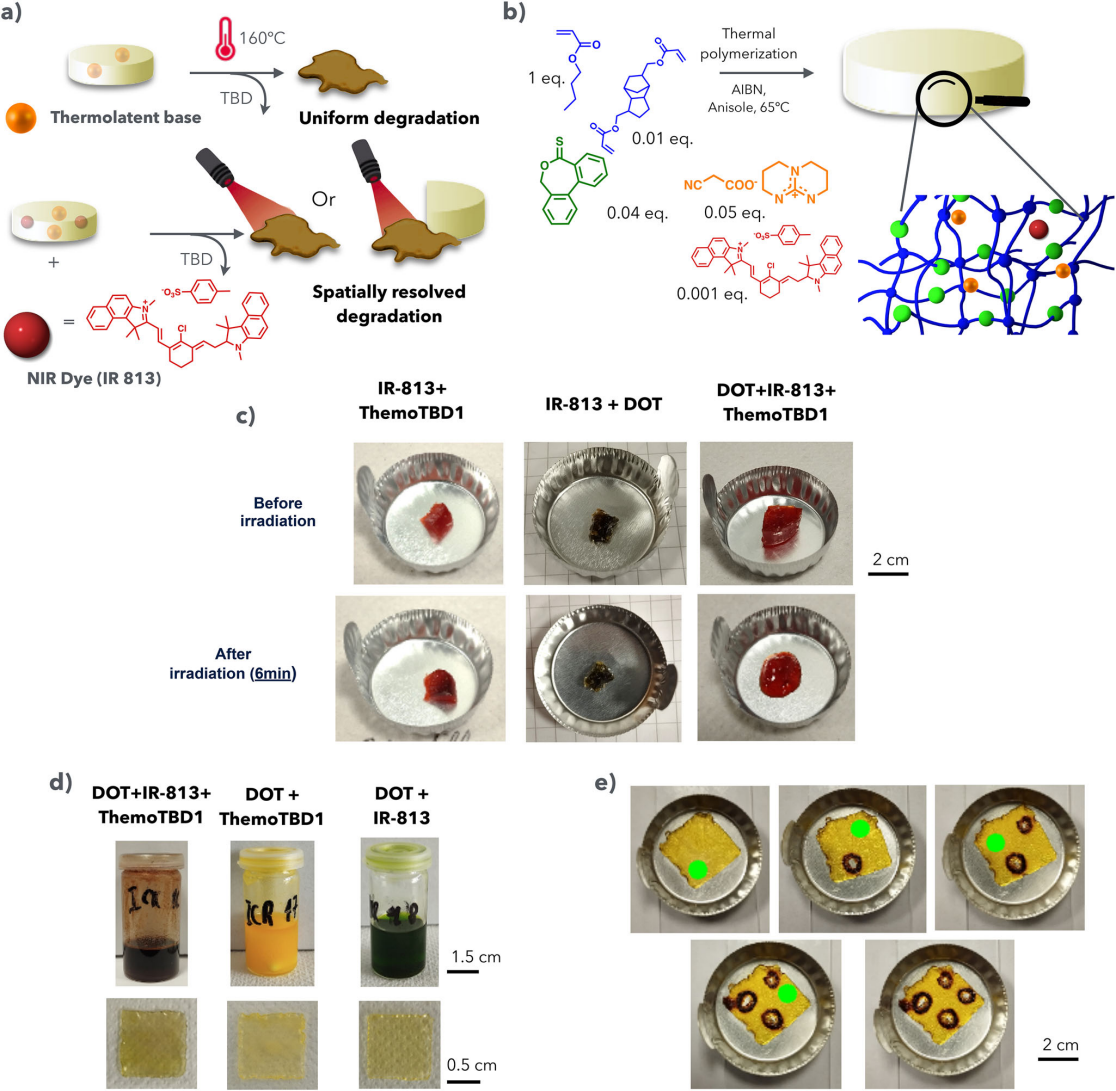
(a) Self‐destruct thermosets using thermally initiated radical ring‐opening copolymerization of DOT and vinyl monomers and containing a NIR heater to make spatially‐resolved degradation triggered by NIR irradiation. (b) Formulation used to prepare such materials. (c) *n*‐Butyl acrylate network containing DOT and/or thermolatent ThermoTBD1 and IR813 before and after irradiating for 6 min with a laser at 808 nm (20 W) of intensity (Table S4). (d) Preparation of thin films by photopolymerization (DLP) at 365 nm (10 mW/cm^2^) of 2 wt% TcddA + 88.9 wt% nBA + 3 wt% DOT + 3 wt% ThermoTBD1 + 3 wt% TPO‐L + 0.1 wt% IR‐813. (e) Spatial resolution of the degradation obtained using a laser irradiation (NIR laser, 808 nm, 37 W/cm^2^). The green dot corresponds to the NIR beam size.

Spatial control is then demonstrated using 250 µm‐thick films photopolymerized from the same formulation (Figure 7d). Localized NIR irradiation produces well‐defined holes whose diameters match the focalized beam size after only a few minutes (Figure 7e). Control films containing IR813 alone or IR813 with ThermoTBD1 show no degradation under identical conditions. This proof‐of‐concept establishes spatially resolved, on‐demand deconstruction of thermosets via NIR photothermal activation, paving the way for selective material removal, patterned recycling, and the development of adaptive high‐performance structures.

### 2.5 End‐of‐Life of the Programmed Deconstructable Thermoset

One of the main challenges for polymer chemists remains the development of high‐performance plastics that combine excellent mechanical and functional properties during service life with complete end‐of‐life management [1, 2]. Two primary EOL scenarios exist. The preferred pathway, recycling (mechanical, chemical, or closed‐loop recycling) conserves energy and reduces reliance on virgin fossil resources, aligning with the principles of a circular plastic economy. However, recycling is often impractical for short‐lifespan or single‐use items (e.g., food packaging) and for applications where collection is impossible. In such cases, biodegradable polymers capable of bio‐assimilation and mineralization by microorganisms in natural environments (soil, oceans) or controlled composting facilities offer a viable alternative. Although End‐of‐Life management is not the primary focus of this study, the thermally triggered deconstruction strategy presented herein generates soluble branched oligomers that hold significant promise for sustainable EOL implementation. The following demonstrations illustrate the potential for chemical recycling or downcycling of these degradation products.

First, we focus on the networks incorporating backbone thioester moieties prepared by radical polymerization, as presented in the previous sections. Only a few studies have addressed the recycling of radically polymerized thermosets [32]. Among them, Reineke and coworkers [33] have demonstrated mechanical recycling of conventional (non‐degradable) thermosets via cryomilling to produce a fine powder from cured parts, which can be reincorporated into fresh liquid monomer resin at loadings up to 30 wt% (with a maximum of 10 wt% for producing 3D‐printed parts comparable to neat analogues) [33]. In contrast, the present approach leverages the inherent solubility of the oligomers produced by backbone cleavage, eliminating the need for energy‐intensive mechanical preprocessing. Oligomers recovered from the thermal deconstruction of Network B were blended at 50 wt% with fresh resin lacking DOT and ThermoTBD1, followed by a second radical polymerization cycle (Figure 8a). The resulting pellet exhibited thermal properties (DSC and TGA) nearly identical to the virgin material (Figure 8b). Dynamic mechanical analysis (DMA) (Figure S6) indicated that the recycled chains primarily act as non‐reactive but well soluble diluent rather than being covalently integrated into the network, consistent with literature reports [17] highlighting the need for end‐group functionalization to achieve full covalent reincorporation. This work is currently being investigated in our lab.

**FIGURE 8:**
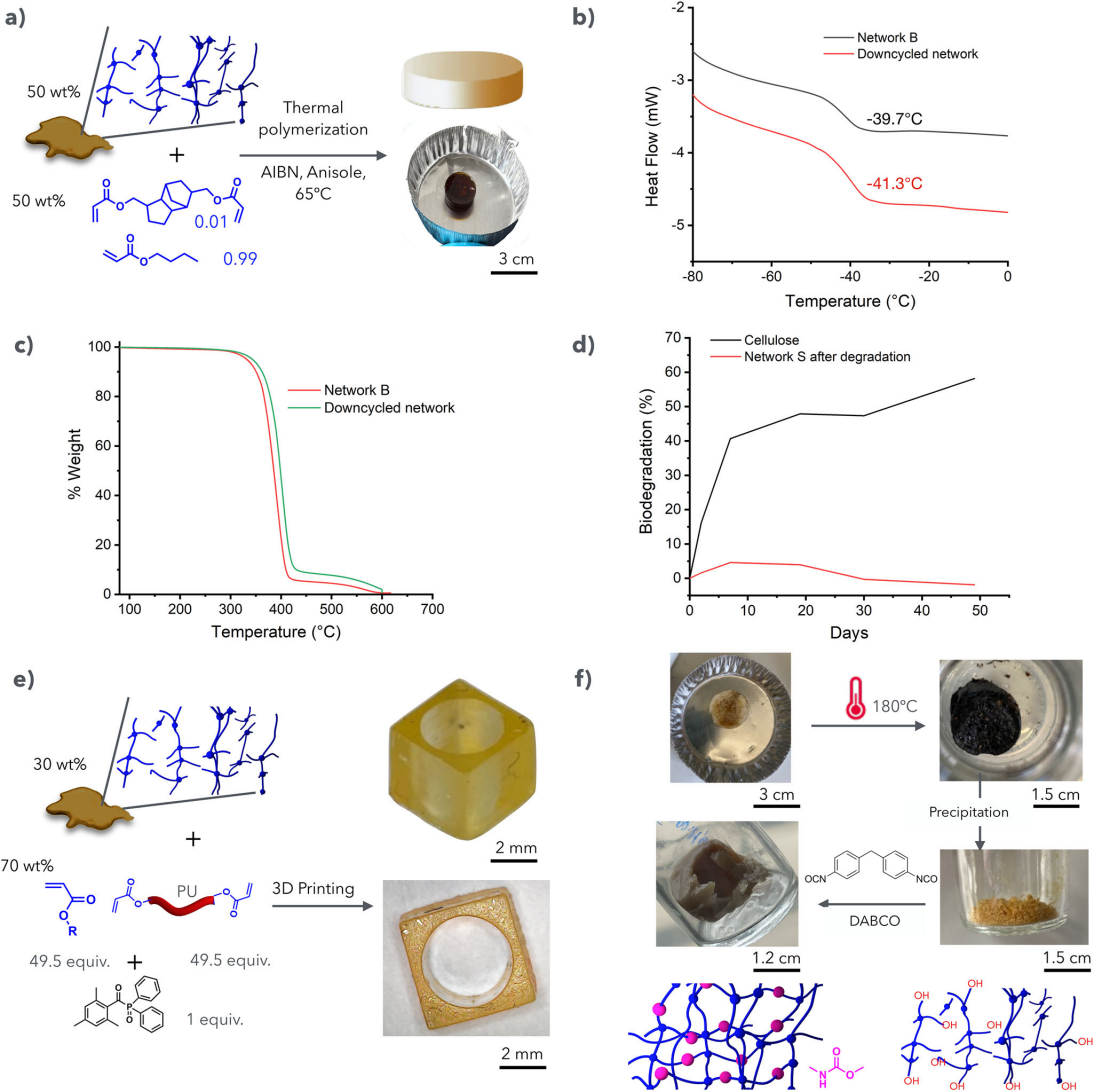
(a) Downcycling experiment using polyacrylate‐based oligomers embedded into a new thermally cured resin. (b) DSC and (c) TGA analyses of the reference polyacrylate network and the downcycled network. (d) Biodegradation kinetic of the sample by monitoring carbon dioxide release under controlled industrial composting conditions, in accordance with ISO 14855‐1. (e) Recycling experiment using a polyacrylate‐based network oligomers coming from the thermal degradation of a 3D object (Resin 4, Section 2.3) used as filler for preparing a new 3D object via vat photopolymerization. (f) Upcycling of PDCPD oligomers to prepare a new polyurethane‐based thermoset following the procedure of Johnson and Moore [19].

Nevertheless, these results validate the feasibility of reincorporating the soluble oligomers into new thermoset materials. This recycling potential is particularly attractive for vat‐photopolymerized networks (Section 2.3). Oligomers obtained from the depolymerization of resin formulation 3 were blended at 30 wt% into fresh resin and subjected to a second photopolymerization cycle. Remarkably, the resulting material maintained excellent printability and high resolution (Figure 8d), demonstrating that a substantial fraction of oligomer waste can be directly reprocessed into new high‐quality photopolymerized objects.

To assess the potential for ultimate aerobic biodegradation of the soluble branched polyacrylate‐based oligomers, biodegradation tests were performed according to ISO 14855‐1 (2012) [34], which specifies the determination of the ultimate aerobic biodegradability of plastic materials under controlled composting conditions. This standard was selected in preference to OECD 301 series tests because ISO 14855‐1 is specifically designed for solid or poorly water‐soluble polymeric materials and oligomers, simulating realistic EOL thermophilic composting conditions (58 ± 2°C, mature compost inoculum)—a more relevant scenario for these polymers than the aqueous activated sludge medium used as inoculum in OECD 301 tests (e.g., 301B)—with direct quantification of CO_2_ evolution. The tests conducted on oligomers derived from Network B yielded negative results (Figure 8c): no significant change in CO_2_ attributable to the tested material was observed over the 50‐day period (unlike the reference cellulose, which biodegrades), indicating that these branched polyacrylate oligomers cannot be assimilated by microorganisms under controlled industrial composting conditions within the timeframe specified by the standard. These findings highlight the need for further structural modifications (e.g., increased hydrophilicity or chain‐end functionalization) to enhance the biodegradability of these degradation products under EOL composting conditions. Notably, the biodegradability of polyacrylate oligomers appears highly sensitive to the alkyl side chain. This is consistent with the findings of Olsen and coworkers [35], who showed that poly(butyl acrylate) is essentially recalcitrant to biodegradation, while the analogous poly(methyl acrylate) undergoes significant microbial degradation. These results emphasize the possibility of tuning biodegradability through simple modification of the acrylate monomer structure.

Lastly, the recycling potential of ROMP‐based thermosets was investigated. This issue regarding the nature of the chain ends has already been extensively addressed by Johnson and coworkers [16, 17, 19], who demonstrated that the hydroxy end‐groups generated upon classic external deconstruction can be advantageously exploited to prepare polyurethane materials by reaction with a multifunctional isocyanate. A key question concerns the nature of the chain ends generated upon deconstruction using the thermolatent approach. To address this, a polysilyl ether model homopolymer was first synthesized by the polycondensation of dichlorodiisopropylsilane and *cis*‐2‐butene‐1,4‐diol (Scheme S1) and subjected to base‐mediated degradation using TBD. Electrospray ionization (ESI)‐mass spectrometry analysis of the resulting oligomers confirmed the formation of telechelic species bearing two hydroxy end‐groups, consistent with the silyl ether cleavage mechanism (Figures S6, S7, Scheme S2, and Table S5). This end‐group functionality mirrors that obtained from conventional degradation (acidic cleavage, TBAF cleavage, and FeCl_3_ in MeOH cleavage), enabling the adoption of established chemical recycling protocols.

Following the upcycling strategy reported by Johnson, Moore and coworkers [19], the telechelic oligomers were reacted with 4,4'‐methylenebis(phenyl isocyanate) (MDI) in the presence of DABCO as catalyst to afford a new polyurethane thermoset (Figure 8e). No extensive characterization of the resulting material was performed, as it was structurally analogous to the polyurethane previously described by Johnson and Moore [19], with comparable thermomechanical properties expected.

Overall, this internal deconstruction platform, applicable across radical, ROMP, and vat‐photopolymerization chemistries, generates well‐defined, soluble oligomers amenable to downcycling, chemical recycling, and upcycling into value‐added materials such as polyurethanes. By eliminating energy‐intensive preprocessing steps and enabling tunable degradation thresholds, the strategy paves the way toward truly circular, high‐performance thermosets with built‐in end‐of‐life programmability, advancing sustainable manufacturing in additive and composite technologies.

### 2.6 Demonstrations of On‐Demand Release and Thermal Sensing Applications

The thermolatent base‐triggered internal depolymerization strategy developed herein enables programmable, solvent‐free degradation of thermosets at predefined temperatures, offering a versatile platform for end‐of‐life management, selective material removal, and integration into functional devices. By combining cleavable comonomers with thermally activated strong bases embedded during curing, the approach achieves rapid network disassembly into soluble oligomers while preserving ambient stability and compatibility with diverse processing methods (radical polymerization, ROMP, vat photopolymerization). This opens broad prospects for applications ranging from on‐demand release of embedded components, spatially patterned recycling, and adaptive or repairable structures to smart sensing devices that respond irreversibly to thermal overloads.

Two representative demonstrations highlight this potential. First, the strategy facilitates the clean thermal recovery of embedded 3D‐printed objects from a degradable thermoset matrix. Building on prior multimaterial DLP‐UV printing concepts [21], where degradable PETIA/DOT domains allowed selective recovery of stable PETIA features (e.g., ghosts and spheres) after external basic treatment, thermal activation provides a direct alternative without chemical immersion.

As an example of the use of thermolatent base, a high‐resolution PETIA David statue (an iconic Marseille landmark) is printed, post‐cured, and encapsulated within the *n*‐butyl acrylate thermoset formulation (*n*BA:TcddA:DOT:ThermoTBD1:AIBN = 1:0.01:0.04:0.05:0.01) via thermal curing at 65°C for 24 h (Figure 9a). Heating the resulting stone‐like object at 160°C for 3 h triggers matrix liquefaction, exposing the intact statue. Subsequent THF washing removes residual thermoset, yielding the pristine embedded object free of contaminants (Figure 9a).

**FIGURE 9:**
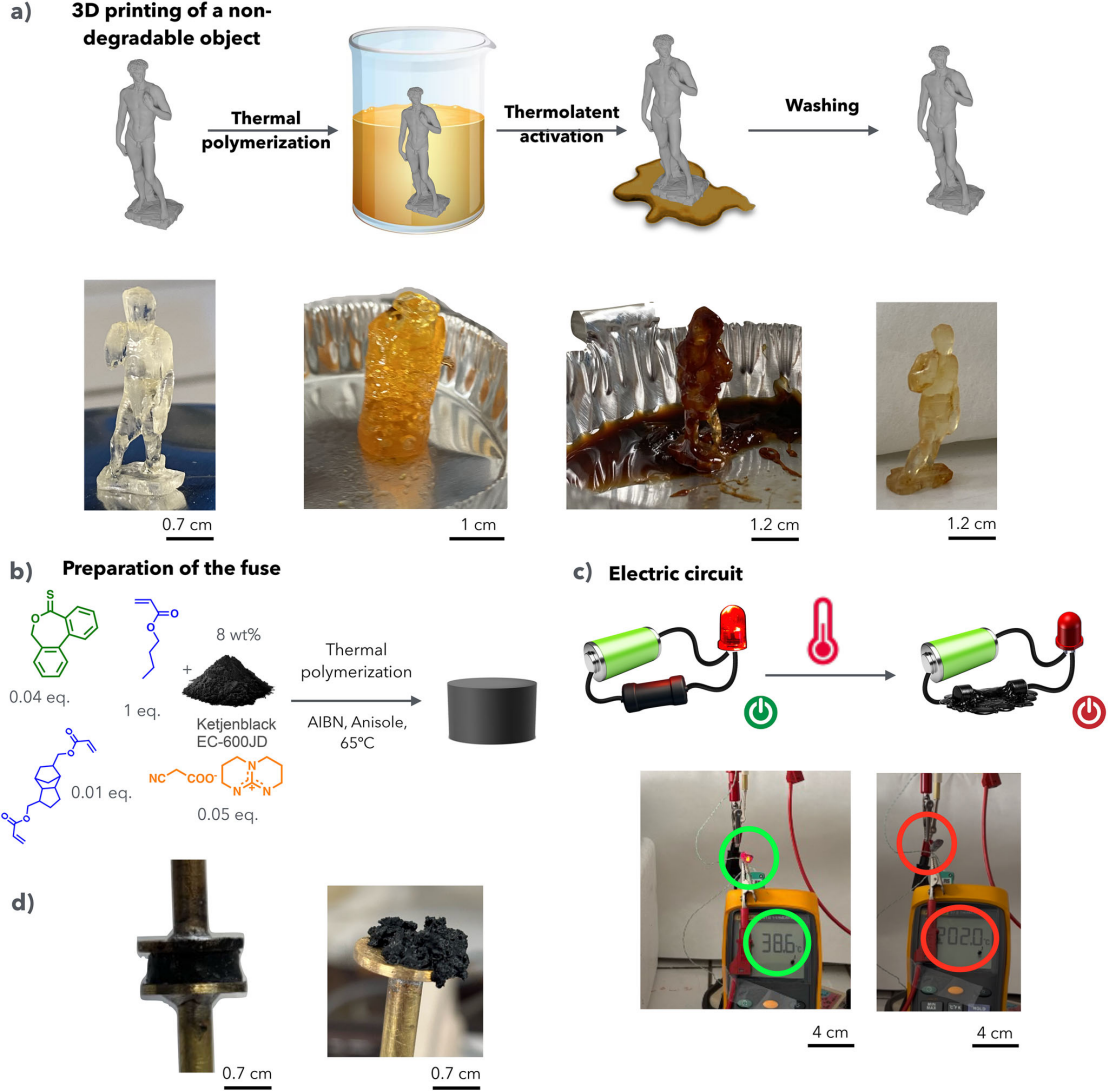
(a) 3D printing of the David statue by VAT photopolymerization using a PETIA/BAPO resin. The 3D object was then immersed in a vial containing a resin *n*BA:TcddA:DOT:ThermoTBD1:AIBN = 1:0.01:0.04:0.05:0.01. After degazing the vial and heating at 65°C for 24 h, a stone like object was recovered after breaking the vial. This object was then heated up to 160°C for 3 h, allowing to recover the David statue that was then washed by immersion in THF. (b) All‐organic fuse prepared using a resin *n*BA:TcddA:DOT:ThermoTBD1:AIBN = 1:0.01:0.04:0.05:0.01 and additional 8 wt% of Ketjenblack EC‐600JD. (c) Concept and experimental validation of the all‐organic fuse. After heating the fuse above 200°C, the electric connection is broken. (d) Pictures of the all‐organic fuse before and after heating.

A second application targets all‐organic thermal fuses, that are temperature‐responsive indicators that interrupt electrical circuits upon overheating, independent of current unless it induces excessive heat. Unlike conventional fuses or circuit breakers, these materials maintain conductivity at ambient conditions but undergo irreversible shape change above a tunable threshold. Conductive networks are fabricated by incorporating additional 8 wt% Ketjenblack EC‐600JD into the same *n*BA:TcddA:DOT:ThermoTBD1:AIBN resin (cured at 65°C for 24 h), with a ThermoTBD1‐free reference as control. Black pellets are sandwiched between conductive plates in a simple circuit powering a red LED (Figure 9c). The filled network sustains sufficient conductivity to illuminate the LED continuously (Video S1). In a controlled heating setup within a glass tube equipped with a resistive wire and thermocouple (Figures 9c and S9), the LED remains lit below 180°C but extinguishes irreversibly between 190°C and 200°C (Figure 9d and Video S2). Post‐examination confirms melting‐induced loss of contact between the plates. Tunability of the activation temperature is readily achieved by varying the thermolatent base, establishing a flexible platform for high‐temperature thermal indicators in electronics, sensors, or safety systems.

## 3 Conclusion

Thermosets, owing to their permanently crosslinked networks, remain inherently non‐recyclable, non‐reprocessable, and resistant to facile degradation, posing significant challenges for end‐of‐life management in a circular economy. While the comonomer approach, i.e. introducing cleavable bonds selectively into polymer backbones, has enabled chemical degradation upon exposure to external acidic or basic solutions, practical implementation is often limited by slow diffusion kinetics and the need for harsh, and hazardous conditions for operators. Here, a transformative strategy is presented: the integration of cleavable comonomers with stimuli‐responsive thermolatent bases directly into the network prior to polymerization. This internal activation mechanism enables programmable, on‐demand deconstruction triggered by heat or near‐infrared (NIR) photothermal stimuli, yielding soluble branched oligomers in appropriate solvents without requiring aggressive external reagents during service life.

The approach is demonstrated across diverse chemistries and processing methods. Radical copolymerization of dibenzo[*c*,*e*]oxepine‐5‐thione (DOT) with styrene, acrylic, and acrylamide monomers, as well as ring‐opening metathesis polymerization (ROMP) of dicyclopentadiene (DCPD) with silyl ether‐containing cyclic olefins, both incorporate tailored thermolatent 1,5,7‐triazabicyclo[4.4.0]dec‐5‐ene (TBD) derivatives. Structural variation of the thermolatent bases allows precise tuning of the activation threshold from ≈120°C to 200°C. Compatibility with thermal and photochemical curing, including vat photopolymerization 3D printing, is preserved, while NIR photothermal triggering imparts spatial resolution to the degradation process.

Practical utility is illustrated through two representative applications: thermally triggered release of an embedded 3D‐printed object from a degradable thermoset matrix, providing a clean, solvent‐free alternative to external degradation; and the realization of all‐organic thermal fuses that maintain electrical conductivity until irreversible melting disrupts contact above a tunable temperature threshold (190°C–200°C in the demonstrated case).

This versatile, stimuli‐responsive platform establishes a general route to high‐performance thermosets with built‐in, programmable degradation, compatible with established manufacturing techniques and offering tunability across chemistries. By addressing key limitations of conventional approaches, the strategy paves the way toward more circular, intelligent polymeric materials for applications in adaptive structures, selective recycling, on‐demand disassembly, and advanced sensing or safety devices.

## Author Contributions

**Sophia Kouider**: investigation, methodology, writing – original draft. **Loic Buchon**: investigation, methodology, writing – original draft, visualization. **Luna Choulot**: investigation, writing – original draft, methodology, visualization. **Paul Greuet**: investigation, writing – original draft, visualization, methodology. **Julie Bratasanu**: investigation, writing – original draft, methodology, visualization. **Patrick Désirée**: investigation, writing – original draft, visualization, methodology. **Jessica Mauriello**: investigation, writing – original draft, methodology. **Elsa Maarek**: investigation, methodology. **Jean‐Louis Clément**: investigation, methodology, visualization. **Laurence Charles**: investigation, writing – original draft, methodology, visualization. **Emmanuelle Gastaldi**: writing – original draft, validation, methodology, visualization, investigation, supervision. **Damien Montarnal**: investigation, writing – original draft, methodology, validation, visualization, supervision. **Olivier Soppera**: investigation, writing – original draft, validation, methodology, visualization, supervision. **Didier Gigmes**: writing – original draft, validation, methodology, visualization. **Catherine Lefay**: conceptualization, investigation, writing – original draft, methodology, validation, visualization, writing – review and editing, supervision. **Yohann Guillaneuf**: project administration, supervision, writing – review and editing, writing – original draft, funding acquisition, investigation, conceptualization, methodology, validation, visualization.

## Conflicts of Interest

S.K., L.B., C.L., O.S. and Y.G. are named inventors on patent (EP25306013.1) filed by the SATT Conectus on the method described in this work.

## Supporting information



Detailed experimental procedures and instrumentation can be found in Supporting Information. The authors have cited additional references within the Supporting Information [36].**Supporting File 1**: anie73962‐sup‐0001‐SuppMat.docx.

**Supporting File 2**: anie73962‐sup‐0002‐MovieS1.mov.

**Supporting File 3**: anie73962‐sup‐0003‐MovieS2.mp4.

## Data Availability

The data that support the findings of this study are available from the corresponding author upon reasonable request.
