# Supplementary material for: On‐Demand Deconstructable Thermosets Through Cleavable Comonomer and Thermolatent Base Synergy
Source: Angew Chem Int Ed Engl. 2026 Aug 6;65(41):e6855232. doi: 10.1002/anie.6855232 (PMC13638089; doi:10.1002/anie.6855232)
Supplement: Supplementary file 1 [file ANIE-65-e6855232-s001.docx]

Supporting Information

On-Demand Deconstructable Thermosets through Cleavable Comonomer and Thermolatent Base Synergy

*Sophia Kouider, Loic Buchon, Luna Choulot, Paul Greuet, Julie Bratasanu, Patrick Désirée, Jessica Mauriello, Elsa Maarek, Jean-Louis Clément, Laurence Charles, Emmanuelle Gastaldi, Damien Montarnal, Olivier Soppera, Didier Gigmes, Catherine Lefay, Yohann Guillaneuf**

S. Kouider, Luna Choulot, Dr. P. Désirée, Dr. J. Mauriello, E. Marek, Dr. J.-L. Clément, Dr. Pr. L. Charles, Dr. D. Gigmes, Dr. C. Lefay, Dr. Y. Guillaneuf

Aix-Marseille Université, CNRS, Institut de Chimie Radicalaire UMR 7273, 13397 Marseille, France
E-mail: (yohann.guillaneuf@univ-amu.fr)

Dr. L. Buchon, Dr. P. Désirée, Dr. O. Soppera

Université de Haute-Alsace CNRS, IS2M UMR 7361, 68100 Mulhouse, France

Université de Strasbourg, 67000 Strasbourg, France

Dr. J. Bratasanu, Dr. D. Montarnal

Univ. Lyon, CPE Lyon, CNRS, Catalysis, Polymerization, Processes and Materials, UMR 5128, F-69003 Lyon, France

P, Greuet, Dr. Emmanuelle Gastaldi

UMR IATE, Université de Montpellier, INRAE, L’Institut Agro Montpellier, 34000, Montpellier, France

***Materials***

n-Butyl acrylate (n-BA, 99%), anisole (99%), azobisisobutyronitrile (AIBN, 97%), 1,5,7-triazabicyclo[4.4.0]dec-5-ene (TBD, 98%), 2-cyanoacetic acid (98%), tricyclo[5.2.1.02,6]decanedimethanol diacrylate (TcddA), isobornyl acrylate (IBA), dicyclopentadiene (DCPD), divinylbenzene (technical grade, 80%) and styrene (≥99%) were purchased from Sigma-Aldrich. 2-Cyano-2-methylpropanoic acid (98%) was purchased from BLDpharm. Diphenyl(2,4,6-trimethylbenzoyl)phosphine oxide (TPO, >98%) and IR-813 *p*-toluenesulfonate (>98%) were purchased from TCI. Tetrahydrofuran [THF, gel permeation chromatography (GPC) grade] was purchased from Fisher. All chemicals purchased from the manufacturers were stored at the recommended temperatures and used as received.

Dibenzo[c,e]oxepane-5-thione (DOT) was synthesized according to the previously published procedure.^1^ **iPrSi-7** and **bisSi-7** were synthesized according to the previously published procedure^2,3^.

*NMR spectroscopy*

^1^H and ^13^C spectra were obtained on a Bruker Avance DPX 400 MHz spectrometer. Tetramethylsilane (TMS) was used as internal standard. The ^1^H chemical shifts were referenced to the solvent peak for CDCl_3_ (δ = 7.26 ppm).

*Size Exclusion Chromatography (SEC)*

SEC analyses were performed using an EcoSEC system from TOSOH equipped with a differential refractometer detector. THF was used as an eluent with 0.25 vol% toluene as a flow marker at a flow rate of 0.3 mL min^−1^ after filtration using Alltech PTFE membranes with a porosity of 0.2 µm. The column oven was kept at 40 °C, and the injection volume was 20 µL. One ResiPore Pre-column (50 mm, 4.6 mm) and two ResiPore columns (250 mm, 4.9 mm) from Polymer Laboratories were used in series. The system was calibrated using polystyrene (PS) standards in the range 100 - 400 000 g.mol^-1^, purchased from Agilent.

*Differential Scanning Calorimetry (DSC).*

Differential scanning calorimetry (DSC) analyses were performed on a TA Instruments (DSCQ20) with the following program (heat-cool-heat cycle): heat from 25 °C to 200 °C at 10 °C.min^–1^, cool to -50 °C at 5 °C.min^–1^ and heat to 200 °C at 10 °C.min^–1^. Heat curves from the second hea/ng are used.

*Mass Spectrometry*

Matrix-assisted laser desorption ionization time-of-flight mass spectrometry (MALDI–TOF-MS) experiments conducted to assess TBD degradation of the linear PBA model were performed with a Bruker UltrafleXtrem mass spectrometer (Bruker Daltonics, Bremen, Germany) operated in the reflectron mode. Norharmane (NOR) was used as the matrix and lithium chloride as the salt. Stock solutions were prepared in as follows: NOR, 0.1 mol.L⁻¹ in THF; LiCl, 0.1 mol.L⁻¹ in methanol; polymer sample, 5 mmol.mL⁻¹ in THF based on SEC data indicating *M*_n_ = 3000 g.mol⁻¹. The stock solutions were combined to achieve a matrix/salt/polymer molar ratio of 1000/100/1 and 2 μL of the ternary mixture was left to air dry on the MALDI plate according to the dried droplet method. The same 40% laser fluence was used for all samples. FlexControl software version 3.4 (Bruker Daltonics) was used for instrument control and data acquisition, and FlexAnalysis software version 3.4 (Bruker Daltonics) was used for data processing.

Electrospray ionization mass spectrometry (ESI-MS) experiments conducted to study degradation products of ROMP-based thermosets were performed with a ZenoTOF mass spectrometer (Sciex, Concord, Ontario, Canada). Samples were dissolved in 500 µL of CH_2_Cl_2_, further diluted (1/1000) in a methanolic solution of sodium chloride (0.1 mM) and introduced in the ESI source with a syringe pump at a 10 μL min^–1^ flow rate. The ESI source was operated under a nebulizing gas (air, 20 psi) and heated at 35°C using the following settings: negative mode, capillary voltage: –4.8 kV, cone voltage: –75 V; positive mode, capillary voltage: +5.5 kV, cone voltage: +75 V. In this hybrid instrument, ions were measured using an orthogonal acceleration time-of-flight (oa-TOF) mass analyzer. In the MS/MS mode, a quadrupole was used for selection of precursor ions to be further submitted to collision-induced dissociation (CID) in a collision cell filled with N_2_. Accurate mass measurements were performed using internal calibration. Instrument control, data acquisition and data processing of all experiments were achieved using Sciex OS software (3.4.0) provided by Sciex.

*Biodegradation*

Respirometric biodegradation tests were performed in a miniaturized set-up as previously described by Salomez et al. (2019), under standardized industrial composting conditions according to ISO 14855-1:2012.  The carbon content of the tested material was determined by elemental analysis (ThermoQuest NA 2500, CE Instruments Ltd, Wigan, UK). Experiments were conducted at 58 ± 1 °C in hermetically sealed glass jars (1 L, Le Parfait, Villeurbanne, France) containing three open polypropylene vials (60 mL each): (i) a vial with 3 g (wet basis) of mature compost (SCH, Aspiran, France) mixed with the tested material corresponding to 50 mg of equivalent carbon, (ii) a vial containing 15 mL of NaOH (0.2 M) to trap CO_2_ released by microbial activity, and (iii) a vial containing distilled water maintain relative humidity close to 100 %.

At regular time intervals, the glass jars were opened and the CO_2_ trapped by the NaOH solution was quantified by back-titration after precipitation of carbonate ions with 5 mL of BaCl₂ solution (20 wt% in water), using thymolphthalein (0.10 % in ethanol) as indicator and HCl (0.1 M) as titrant. The compost vial was weighed, and water was added when necessary to maintain 50 % moisture content. After each measurement, the NaOH vial was replaced with fresh solution, the jars were aerated for 2 min, sealed, and returned to the incubator.

Biodegradation tests included a positive control (cellulose powder, Arbocel BE 600–10 TG grade, d₅₀ = 18 µm) and a blank (compost without test material). All experiments were performed in triplicate, and CO₂ production measured in the blank was subtracted from that of the test samples. The degree of biodegradation (DEG, %) was calculated as:

$$DEG \left( \% \right)= \frac{{CO}_{2, Material}- {CO}_{2, Blank}}{{CO}_{2 max}} \times100$$

with

$${CO}_{2 max}= C \times\frac{44.01}{12.01}$$

where CO₂,_Material_ and CO₂,_Blank_ are the amounts of CO₂ produced (mg) in the test and blank jars, respectively, and C is the amount of carbon introduced with the test material (mg).

*Thermogravimetric analysis (TGA)*

Thermogravimetric analyses were performed using a PerkinElmer 8000 instrument with a temperature ramp from 40 to 800 °C at a heating rate of 10 °C.min^−1^ , under a nitrogen atmosphere with a flow rate of 20 mL min⁻¹.

*Rheological characterization*

Rheology experiments were performed on a MARS 60 apparatus from ThermoScientific using a Peltier oven and parallel plate geometries of 8 mm diameter. The networks were prepared in flat vials (25 mm diameter and ca. 1.5 mm high films), then recovered and thoroughly dried (ultra high vacuum around 10^-7^ mbar, 50°C, 24h) to eliminate any residual solvent or monomer. 8 mm disks were punched from the films and equilibrated at 50°C in the rheometer before analysis. Temperature sweeps from 25°C to 180°C (3K min^-1^) were carried while applying small amplitude oscillatory shear (SAOS, 1% strain, 1 Hz). Frequency sweeps at 30°C (1% strain) were acquired before and after the temperature sweep to quantify the evolution of viscolelastic properties.

*Near Infrared laser*

Degradations of polymer samples under infrared irradiation were performed using a Coherent Compact SE Series continuous near-infra-red (808 nm) laser. Irradiation intensity was set at 20 W.cm^−2^. The samples to be degraded were placed 15 cm from the laser output.

*3D printing*

3D printing was performed using a MiiCraft Ultra 125Y DLP 3D printer, capable of printing successive polymer layers with thicknesses ranging from 5 to 500 µm, using a 385 nm wavelength light source and the associated software named Ultimaker. The layer thickness was set to 50 µm.

***General Procedure for thermolatent salts.***

Cyanoacetic acid or 2-cyano-2-methylpropanoic acid (9.99 mmol, 1 eq) and 1,5,7-triazabicyclo[4.4.0]dec-5-ene (TBD) or 1,1,3,3-tetramethylguanidine (TMG) (9.99 mmol, 1 eq) were dissolved in 40 mL of chloroform. After stirring overnight at room temperature, the chloroform was removed using a rotary evaporator to obtain a white powder (quant.).

**ThermoTBD1**: ^1^H NMR (400 MHz, CDCl_3_, δ): 1.93-1.99 (quint, J=5.95Hz, 4H); 3.23-3.28 (m, 10H); 10.04 (s, 2H)

**ThermoTBD2**: ^1^H NMR (400 MHz, CDCl_3_, δ): 1.52 (s, 6H); 1.95 (quint, J= 5.86 Hz, 4H); 3.22 (t, J=6.01, 4H); 3.28 (m, 4H); 10.23 (s, 2H). ^13^C NMR (100 MHz, CDCl_3_, δ): 175.85 (COO-); 151.69 (N-C-NH_2_); 124.93 (CN); 46.91 (N-CH_2_); 41.18 (C); 37.80 (2*CH_2_); 25.66 (2*CH_2_); 20.93 (2*CH_2_)

HRMS (ESI, [M]^+^) calcd for [C_7_H_14_N_3_] ^+^: *m/z* 140.1182 found: *m/z* 140.1184 Da.

HRMS (ESI, [M]^-^) calcd for [C_5_H_6_NO_2_]^-^: *m/z* 112.0404 found: *m/z* 112.0409.

**ThermoTMG**: ^1^H NMR (400 MHz, CDCl_3_, δ): 2.95 (s, 12H); 3.22 (s, 2H); 8.71 (s, 2H).

^13^C NMR (100 MHz, CDCl_3_, δ): 27.44 (CH_2_-CN); 39.74 (4*CH_3_); 117.88 (CN); 162.35 (CNH_2_^+^); 166.58 (COO^-^)

HRMS (ESI, [M]^+^) calcd for [C_5_H_14_N_3_] ^+^: *m/z* 116.1182 found: *m/z* 116.1185.

HRMS (ESI, [M]^-^) calcd for [C_3_H_2_NO_2_]^-^: *m/z* 84.0091 found: *m/z* 84.0097.

***General Procedure for Gel Synthesis via rROP.***

AIBN (0.070 mmol, 0.01eq), n-butyl acrylate (n-BA) or isobornyl acrylate (IBA) or styrene (S) or N,N-dimethylacrylamide (DMAA) (7.02 mmol, 1 eq), anisole (1 mL), dibenzo[c,e]oxepane-5-thione (DOT) (0−0.28 mmol, 0-0.04 eq), thermolatent(0-0.35 mmol, 0-0.05eq) and tricyclo[5.2.1.02,6]decanedimethanol diacrylate (TcddA) as cross-linker (divinyl benzene or NN'-methylene-bis-acrylamide for copolymerization with styrene and N,N-dimethylacrylamide) (0.070 mmol, 0.01eq) were added to a neckless glass vial and stirred until the reaction mixture was homogenous. The stirrer was removed, and the vial was sealed with a septum and degassed with nitrogen for 10 min before heating at 65 °C for 24 h.

**Table S1.** Description of synthesis recipes for networks (A-T) prepared by thermal radical polymerization

| **Materials** | **n-BA** | **IBA** | **S** | **DMA** | **DOT** | **Thermolatent** | **Difunctional monomer** | **Anisole** | **AIBN** |
| --- | --- | --- | --- | --- | --- | --- | --- | --- | --- |
| **A** | 7.02 mmol - 1 eq | 0 | 0 | 0 | 0 | 0 | **TcddA**  0.070 mmol - 0.01 eq | 1 mL | 0.070 mmol  - 0.01eq |
| **B** | 7.02 mmol - 1 eq | 0 | 0 | 0 | 0.28 mmol - 0.04 eq | 0 |  |  |  |
| **C** | 7.02 mmol - 1 eq | 0 | 0 | 0 | 0 | **ThermoTBD1**  0.21 mmol - 0.03eq |  |  |  |
| **D** | 7.02 mmol - 1 eq | 0 | 0 | 0 | 0.28 mmol - 0.04 eq | **ThermoTBD1**  0.21 mmol - 0.03eq |  |  |  |
| **E** | 0 | 7.02 mmol - 1 eq | 0 | 0 | 0 | 0 |  |  |  |
| **F** | 0 | 7.02 mmol - 1 eq | 0 | 0 | 0.28 mmol - 0.04 eq | 0 |  |  |  |
| **G** | 0 | 7.02 mmol - 1 eq | 0 | 0 | 0 | **ThermoTBD1**  0.21 mmol - 0.03eq |  |  |  |
| **H** | 0 | 7.02 mmol - 1 eq | 0 | 0 | 0.28 mmol - 0.04 eq | **ThermoTBD1**  0.21 mmol - 0.03eq |  |  |  |
| **I** | 0 | 0 | 7.02 mmol - 1 eq | 0 | 0 | 0 | **Divinylbenzene**  0.070 mmol - 0.01 eq |  |  |
| **J** | 0 | 0 | 7.02 mmol - 1 eq | 0 | 0.28 mmol - 0.04 eq | 0 |  |  |  |
| **K** | 0 | 0 | 7.02 mmol - 1 eq | 0 | 0 | **ThermoTBD1**  0.21 mmol - 0.03eq |  |  |  |
| **L** | 0 | 0 | 7.02 mmol - 1 eq | 0 | 0.28 mmol - 0.04 eq | **ThermoTBD1**  0.21 mmol - 0.03eq |  |  |  |
| **M** | 0 | 0 | 0 | 7.02 mmol - 1 eq | 0.28 mmol - 0.04 eq | 0 | **NN' Methylene-bis-acrylamide** 0.070 mmol - 0.01 eq |  |  |
| **N** | 0 | 0 | 0 | 7.02 mmol - 1 eq | 0.28 mmol - 0.04 eq | **ThermoTBD1**  0.351 mmol - 0.05eq | **NN' Methylene-bis-acrylamide** 0.070 mmol - 0.01 eq |  |  |
| **O** | 7.02 mmol - 1 eq | 0 | 0 | 0 | 0.28 mmol - 0.04 eq | **ThermoTBD1**  0.07 mmol - 0.01eq | **TcddA**  0.070 mmol - 0.01 eq |  |  |
| **P** | 7.02 mmol - 1 eq | 0 | 0 | 0 | 0.28 mmol - 0.04 eq | **ThermoTBD1**  0.14 mmol - 0.02eq |  |  |  |
| **Q** | 7.02 mmol - 1 eq | 0 | 0 | 0 | 0.28 mmol - 0.04 eq | **ThermoTBD1**  0.28 mmol - 0.04eq |  |  |  |
| **R** | 7.02 mmol - 1 eq | 0 | 0 | 0 | 0.28 mmol - 0.04 eq | **ThermoTBD1**  0.35 mmol - 0.05eq |  |  |  |
| **S** | 7.02 mmol - 1 eq | 0 | 0 | 0 | 0.28 mmol - 0.04 eq | **ThermoTBD2**  0.21 mmol - 0.03eq |  |  |  |
| **T** | 7.02 mmol - 1 eq | 0 | 0 | 0 | 0 | **ThermoTBD2**  0.21 mmol - 0.03eq |  |  |  |

**Figure S1**. Variation of the amount of ThermoTBD1 and SEC analyses of the degraded product. A DOT containing network was degraded using a solution of TBD in THF (2.5 wt%) and was also analyzed by SEC to compare the internal and external degradation.

***Thermal degradation procedure.***

Approximately 1.5 g of each material was subjected to a temperature of 140°C, 200°C, 130°C, 180°C for thermolatent salt **ThermoTBD1**, **ThermoTBD2** or ThermoTMG respectively in an oven for 1-3 hours.

***External degradation of gels***

Approximately 30 mg of a gel sample containing DOT was stirred in 1.5 mL THF and 1.5 mL of 5%wt TBD in THF at RT for 24 h. The sample was concentrated under vacuum. Dichloromethane (CH₂Cl₂) was added, and the organic phase was washed with 6M HCl and water. The organic layer was then dried over sodium sulfate (Na₂SO_4_) and concentrated under vacuum.

Similar degradation was done using 1.5 mL THF and 1.5 mL of n-butylamine at RT for 24 h.

***Degradation of linear P(n-BA-co-5%DOT)***

To better understand the degradation of the cross-linked networks, a linear P(nBA-co-DOT) analogue was synthesized and degraded under same conditions (Figure S2a).

AIBN (0.03mmol, 0.0005 eq), *n*-BA (0.08mol, 1.0 eq), DOT (4mmol, 0.05 eq), and anisole (50 wt% relative to monomers) were introduced into a 50 mL round-bottom flask. The reaction mixture was stirred until homogeneous, degassed with nitrogen for 10 min, and heated at 80 °C for 6 h. Polymerization was quenched by exposure to air, and the resulting polymer was precipitated into cold methanol. The solid was collected and dried under vacuum. The molar mass distribution of the resulting polymer was analyzed by SEC.

Mₙ = 324 kg·mol⁻¹, M_w_ =798 kg·mol⁻¹, and Đ =2.5.

The polymer was subsequently degraded according to the procedure described above in the section “External degradation of gels” for 1 h. The molar mass distribution of the degraded sample was also analyzed by SEC.

Mₙ = 3.2 kg·mol⁻¹, M_w_ =8.3 kg·mol⁻¹, and Đ =2.6.

MALDI–TOF mass spectrometry analyses were performed on the degradation products of the linear P(*n*-BA-co-DOT) model. The MALDI mass spectrum recorded in the positive ion mode (Figure S2c) shows two main distributions of peaks assigned to a PBA polymer adducted to Li^+^, consistent with the use of LiCl as the cationization agent, and also ionized as protonated chains, which suggests one basic end-group. Calculation of end-group masses are consistent with the structure named ^1^P in Figure S2b. The MALDI mass spectrum recorded in the negative ion mode (Figure S2d) shows another PBA ionized as deprotonated chains and measured *m/z* values are consistent with the structure named ^2^P in Figure S2b.

Based on the expected degradation mechanism involving an addition–elimination process, the presence of a TBD group at the α-chain end was anticipated, which is indeed observed. In contrast, a thiol functionality was expected at the ω-chain end; however, no such species is detected. Instead, the ω-chain end corresponds to a sulfonic acid (–SO₃H) moiety, as evidenced by structure ^2^P.

This observation strongly suggests oxidative transformation of the initially formed thiol end-group, most likely promoted by the solvent and the presence of oxygen during degradation. In addition, a second degradation product, denoted ^1^P, is detected and corresponds to a PBA chain terminated by a hydrogen atom. This species can reasonably arise from the elimination of the sulfonic acid group, potentially via transient formation of an alcohol (–OH) end-group followed by dehydration, ultimately leading to a saturated ω-chain end.

Importantly, MALDI–TOF MS analysis of a degraded cross-linked P(n-BA-*co*-DOT) network obtained using **thermoTBD1** as the degradation trigger also reveals the presence of structure ^2^P, detected as Li⁺ adducts in the positive ion mode. In addition to this main distribution, several other polymeric distributions are observed (Figure S2e), reflecting the increased structural heterogeneity inherent to the degradation of a cross-linked network while remaining consistent with the proposed degradation pathway.

**Figure S2.** (a) Mechanism of polymerization of P(nBA-*co*-DOT) and its degradation; (b) Structure proposed for ^1^P and ^2^P polymers detected in MALDI-MS; MALDI mass spectra recorded for the degradation products of a P(n-BA-*co*-DOT) model in (c) the positive ion mode and (d) the negative ion mode. (e) MALDI mass spectrum recorded in the positive ion mode for the degradation products of a cross-linked P(n-BA-*co*-DOT) network.

**Figure S3.** Reference DMA network and network of DMA containing 4 mol% DOT and thermolatent **ThermoTBD1** before and after heating at 160 °C (Table S1 – Network M and N).

**Table S2.** Comparison of the SEC analyses results of the degraded product depending of the nature and amount of thermolatent used. A DOT only containing network was degraded using a solution of TBD in THF (2.5 wt%) and was also analyzed by SEC to compare the internal and external degradation.

| **Thermolatent base (eq)** | **0 (external degradation)** | **ThermoTBD1 (5)** | **ThermoTBD1 (4)** | **ThermoTBD1 (3)** | **ThermoTBD1 (2)** | **ThermoTBD1 (1)** | **ThermoTBD2 (3)** | **ThermoTMG (3)** |
| --- | --- | --- | --- | --- | --- | --- | --- | --- |
| *M*_n_ (g.mol^-1^) | 3900^a^ | 5800 | 5300 | 5600 | 5800 | 4900 | 5800 | 6700 |
| *M*_w_ (g.mol^-1^) | 9250^a^ | 17500 | 14800 | 19900 | 20700 | 16400 | 23500 | 38000 |
| *Đ* | 2.4^a^ | 3.0 | 2.8 | 3.5 | 3.5 | 3.3 | 4.1 | 5.7 |

**Figure S4.** Comparison of the molar mass distribution of the degraded oligomers obtained using different amounts of ThermoTBD1 and with the use of other thermolatent structures.

***General Procedure for Gel Synthesis via ROMP.***

Mixture of DCPD, iPrSi-7 (0.1eq) or bisSi-7 (0.05-0.07 eq) and thermolatent salts (ThermoTBD1 or ThermoTBD2) (0-0.1eq) were added to a vial containing finely powdered Grubbs’ 2nd generation catalyst (2 mg.mL^−1^) and tributyl phosphite (1:1 molar ratio to Grubbs’ 2nd generation catalyst).The mixture was cured in an oven at 120 °C for 15 min. The vials were then broken to release the samples.

***External degradation of gels.***

A gel sample containing **iPrSi-7** or **bisSi-7** was stirred in 10 mL of 2%wt or 1%wt TBD in THF for 24 h. The sample was concentrated under vacuum. Dichloromethane (CH₂Cl₂) was added, and the organic phase was washed with 6M HCl and water. The organic layer was then dried over sodium sulfate (Na₂SO_4_) and concentrated under vacuum.

**Table S3.** Comparison of the SEC analyses results of the degraded ROMP-based oligomers depending on the degradation conditions used.

|  | *M*_n_ (g.mol^-1^) | *M*_w_ (g.mol^-1^) | *Ð* |
| --- | --- | --- | --- |
| P(DCPD-*co*-iPr-Si7)  ext deg | 7,000 | 15,000 | 2.1 |
| P(DCPD-*co*-bis-Si7)  ext deg | 9,500 | 34,000 | 3.6 |
| P(DCPD-*co*-bis-Si7)  **ThermoTBD1** | 11,000 | 28,000 | 2.5 |
| P(DCPD-*co*-bis-Si7)  **ThermoTBD2** | 11,000 | 30,000 | 2.7 |

**Figure S5.** Comparison of the molar mass distribution of the degraded oligomers issued from ROMP networks obtained using different degradation conditions (external degradation using TBD or after heating with either **ThermoTBD1** or **ThermoTBD2**).

***3D-object***

Resin 1: (PETIA + 2wt%DOT + 1wt% TPO) base layers (3*100s) curing time 90 s.

Resin 2: (PETIA + 2wt%DOT + 1wt% TPO+ 5wt%ThermoTBD1) base layers (3*100s) curing time 90 s.

Resin 3: 2% tcddA + 90% IBA + 3% DOT + 5% TL + 1% TPO.

-	For Reference without DOT and ThermoTBD1: 30 s per layer

-	Only thermoTBD1: 40s per layer

-	thermoTBD1 + DOT: 120s per layer

Resin 4: 45.5% diacrylate polyurethane 45.5% monoacrylate. + 3% DOT + 5% TL + 1% TPO -	For Reference without DOT and ThermoTBD1: 3*40s (base layers) curing time 15s

-	Only DOT: 3*200s (base layers) curing time 120s

-	thermoTBD1 + DOT: 3*220s (base layers) curing time 135s

Three degradation conditions were applied (in approximately 20 mL of solvent in each case):

(1) 5 wt% TBD in THF at room temperature for 1 day, followed by 18 hours under reflux.

(2) 5 wt% KOH in THF/MeOH (1:1 v/v) at room temperature for 8 hours.

(3) 50 vol% n-butylamine in THF at room temperature for 1 day, followed by 18 hours under reflux.

***IR-irradiation degradation procedure***

**Preparation of the network**

AIBN (0.070 mmol, 0.01eq), n-BA (7.02 mmol, 1 eq), anisole (1 mL), DOT (0−0.28 mmol, 0-0.04 eq), TcddA (0.070 mmol, 0.01 eq), thermoTBD1 (0-0.21 mmol, 0-0.03eq) and IR813 (0-0.0070 mmol, 0-0.001eq) were added to a neckless glass vial and stirred until the reaction mixture was homogenous. The stirrer was removed, and the vial was sealed with a septum and degassed with nitrogen for 10 min before heating at 65 °C for 24 h.

The samples were then illuminating using a laser irradiating at 808 nm with an intensity of 20W for 6 minutes.

**Table S4.** Description of synthesis recipes for networks prepared by thermal radical polymerization and degraded under photothermal activation

| **Materials** | **n-BA** | **ThermoTBD1** | **DOT** | **IR813** | **TcddA** | **Anisole** | **AIBN** |
| --- | --- | --- | --- | --- | --- | --- | --- |
| **Reference** | 7.02 mmol - 1 eq | 0 | 0 | 0 | 0.070 mmol - 0.01 eq | 1 mL | 0.070 mmol  - 0.01eq |
| **DOT** |  | 0 | 0.28 mmol - 0.04 eq | 0 |  |  |  |
| **IR813+ThermoTBD1** |  | 0.21 mmol - 0.03eq | 0 | 0.0070 mmol - 0.001eq |  |  |  |
| **IR813** |  | 0 | 0 | 0.0070 mmol - 0.001eq |  |  |  |
| **IR813+DOT** |  | 0 | 0.28 mmol - 0.04 eq | 0.0070 mmol - 0.001eq |  |  |  |
| **IR813+ThermoTBD1 + DOT** |  | 0.21 mmol - 0.03eq | 0.28 mmol - 0.04 eq | 0.0070 mmol - 0.001eq |  |  |  |

**Spatially resolved photodegradation**

A thin film network was prepared using a resin composed of 2%wt TcddA + 88,9%wt nBA + 3%wt DOT + 3wt%ThermoTBD1 + 3%wt TPO-L + 0.1 wt% IR-813. The resin was irradiated at 365 nm using a DLP (8100mA; 10mW/cm², 15 min).A thin film of 250 μm was obtained.

Spatial resolution of the degradation obtained using a laser irradiation (NIR laser, 808 nm, 4A, 37 W/cm2).

***End of Life of the programmed deconstructable rROP network***

In a screw-cap glass vial, AIBN (0.07 mmol, 0.01 eq), n-butyl acrylate (n-BA; 7.02 mol, 1.0 eq), tricyclo[5.2.1.0 2,6]decanedimethanol diacrylate (TcddA, cross-linker; 0.070 mmol, 0.01 eq), the gel degradation product D (Table S1) (30 wt% relative to the total monomer mass), and anisole (50 wt% relative to the total monomer mass) were combined. The mixture was stirred until homogeneous, degassed under nitrogen for 10 min, and then heated at 80 °C for 6 h. The polymerization was quenched by exposure to air.

Prior to DMA, DSC, and TGA analyses, anisole was removed by evaporation in a vacuum oven at 150 °C until constant mass.

**Figure S6.** Mechanical analysis (1% strain, 1 Hz, 3 K min^-1^) of initial cleavable *n*-BuA network incorporating DOT (green, Network B) and the downcycled material (50 wt% of oligomers obtained after depolymerization of D and 50 wt% of pristine monomers) (orange).

***End of Life of the programmed deconstructable 3D object***

The oligomers recovered after degradation of Resin 3 using ThermoTBD1 were dried under vacuum and subsequently incorporated (30 wt%) into a photocurable resin composed of a monoacrylate (50 wt%), a diacrylate polyurethane (49 wt%), and TPO (1 wt%). For 3D printing, the base layers were exposed for 3 × 50 s, followed by an exposure time of 35 s per layer.

***Degradation products of the model Silylether polycondensate***

In a round-bottom flask equipped with a magnetic stir bar, cis-2-butene-1,4-diol (0.412 mL, 5 mmol, 1.0 eq) was introduced under an argon atmosphere, followed by imidazole (0.68 g, 10 mmol, 2.0 eq). Dichlorodiisopropylsilane (0.902 mL, 5 mmol, 1.0 eq) was then added dropwise. The reaction mixture was stirred at room temperature for several hours.

The reaction was subsequently quenched by the addition of water (300 mL) and extracted with pentane (2 × 400 mL). The combined organic layers were dried over anhydrous Na₂SO₄, filtered, and concentrated under reduced pressure to afford a colorless oil.

Degradation experiments were performed on model silylether polycondensate samples mimicking a Poly(iPRSi-7) homopolymer (150 mg) in a TBD solution (2.5 wt%, 10 mL). The mixtures were stirred at room temperature for either 2 h or overnight (Scheme S1)

**Scheme S1.** The synthesis of the polycondensate and the expected degradation products

Owing to the small size of molecules expected upon TBD-degradation of silylether polycondensate, this mechanistic study was performed using ESI instead of MALDI as the ionization technique for mass spectrometry analyses. The ESI mass spectrum obtained before degradation displays three main distributions of peaks spaced by the 200.1 Da mass of the silylether polycondensate repeating unit (annotated with circles in Figure S7a). Using *m/z* values of this singly charged sodium adducts to calculate the global mass of end-groups permits to propose structures (Scheme S2) that are supported by accurate mass measurements (Table S5). In Figure S6a, major signals could be assigned to the targeted polymer with m_α_+m_ω_ = 88.1 Da (black circles, with DP = 1-6) while cyclic oligomers with m_α_+m_ω_ = 0 Da (open circles, with DP = 2-5) and H/OH-terminated oligomers with m_α_+m_ω_ = 18.0 Da (grey circles, with DP = 1-7) are most probably synthesis by-products. Two other minor distributions would correspond to degradation products caused by the presence of water in solvents employed for synthesis, with peaks annotated by red triangles in Figure S6a assigned to the expected degradation species with m_α_+m_ω_ = 148.1 Da. Accurate mass measurements (Table S5) also permit to determine elemental composition of secondary degradation products (annotated by purple triangles Figure S7a) and to propose a structure for these oligomers with m_α_+m_ω_ = 278.2 Da.

Samples obtained after TBD degradation were first extracted in CH_2_Cl_2_ to minimize the amount of TBD that was observed to suppress the signal of any other species in positive mode ESI. As compared to Figure S7a, the abundance and length of chains of starting polymers (annotated by circles) decrease after 2 hours of TBD treatment (Figure S7b) and are no longer observed after one night (Figure S7c). On the other hand, degradation products (annotated by triangles) become major, and their chain length decreases as the duration of the TBD treatment is increased from two hours (Figure S6b) to one night (Figure S7c). Although the ultimate degradation product (148.1 Da) with no model *i*PrSi-7 repeating unit was not observed as a sodium adduct (no peak at *m/z* 171.1 in Figure S7a), it is clearly detected as a deprotonated molecule in the negative ion mode (*m/z* 147.1 in Figure S8a). This assignment is supported by accurate mass measurement (C_6_H_15_O_2_Si^–^: *m/z*_th_ 147.0847, *m/z*_exp_ 147.0845) and by MS/MS (Figure S8b).

**Figure S7.** Positive mode ESI mass spectra of linear silylether polycondensate using NaCl as the cationizing agent: a) before degradation, and after degradation with TBD for b) 2 hours and c) 1 night. All species are singly charged sodium adducts. See text and Scheme S1 for symbols. Peaks annotated with italicized *m/z* values do not belong to oligomeric distribution.

**Scheme S2.** Proposed structure for oligomers observed in ESI (+)-MS of linear model silylether polycondensate.

**Table S5.** Accurate mass measurements of sodiated oligomers observed in the positive mode ESI mass spectrum recorded before TBD-degradation of linear model silylether polycondensate, which monomer has an elemental composition of C_10_H_20_O_2_Si (200.1233 Da).

|  | α | ω | DP | composition | *m/z*_th_ | *m/z*_exp_ |
| --- | --- | --- | --- | --- | --- | --- |
|  | H | OCH_2_CH=CHCH_2_OH | 1 | C_14_H_28_O_4_SiNa^+^ | 311.1649 | 311.1651 |
|  |  |  | 2 | C_24_H_48_O_6_Si_2_Na^+^ | 511.2882 | 511.2881 |
|  |  |  | 3 | C_34_H_68_O_8_Si_3_Na^+^ | 711.4114 | 711.4112 |
|  | - | - | 2 | C_20_H_40_O_4_Si_2_Na^+^ | 423.2357 | 423.2358 |
|  |  |  | 3 | C_30_H_60_O_6_Si_3_Na^+^ | 623.3590 | 623.3590 |
|  |  |  | 4 | C_40_H_80_O_8_Si_4_Na^+^ | 823.4823 | 823.4815 |
|  | H | OH | 1 | C_10_H_22_O_3_SiNa^+^ | 241.1230 | 241.1232 |
|  |  |  | 2 | C_20_H_42_O_5_Si_2_Na^+^ | 441.2463 | 441.2464 |
|  |  |  | 3 | C_30_H_62_O_7_Si_3_Na^+^ | 641.3696 | 641.3693 |
|  | HOSi(C_3_H_7_)_2_ | OH | 1 | C_16_H_36_O_4_Si_2_Na^+^ | 371.2044 | 371.2045 |
|  |  |  | 2 | C_26_H_56_O_6_Si_3_Na^+^ | 571.3277 | 571.3274 |
|  |  |  | 3 | C_36_H_76_O_8_Si_4_Na^+^ | 771.4510 | 771.4504 |
|  | HOSi(C_3_H_7_)_2_ | OSi(C_3_H_7_)_2_OH | 0 | C_12_H_30_O_3_Si_2_Na^+^ | 301.1626 | 301.1626 |
|  |  |  | 1 | C_22_H_50_O_5_Si_3_Na^+^ | 501.2858 | 501.2858 |
|  |  |  | 2 | C_32_H_70_O_7_Si_4_Na^+^ | 701.4091 | 701.4080 |

**Figure S8.** a) Negative mode ESI mass spectrum of linear model silylether polycondensate after degradation with TBD for 1 night. Peaks annotated with italicized *m/z* values do not belong to oligomeric distribution. b) MS/MS of the deprotonatd molecule at *m/z* 147.1, with inset table reporting accurate mass measurements of fragments using the precursor ion as an internal standard (I.S.).

***End of Life of the programmed deconstructable ROMP***

Upcycling was performed starting from a material containing 1.75 mol% of bisSi-7. The degraded material (350 mg) was placed in a vial and dissolved in dichloromethane (3.5 mL) under stirring until complete dissolution.

DABCO catalyst (0.009 g, 0.2 equiv. relative to the –OH content) was then added, followed by anhydrous magnesium sulfate (MgSO₄, 5 mg), and the mixture was stirred again. The suspension was filtered to completely remove the solid magnesium sulfate, and dichloromethane (2 mL) was used to rinse the filter and quantitatively transfer the remaining material.

Finally, methylene diphenyl diisocyanate (MDI, 0.05 g, 0.5 equiv. relative to the –OH content) was added, and the reaction mixture was stirred overnight.

***Combination non degradable object and degradable network.***

We prepared a 3D object using a PETIA resin (David Statue). Once the statue has been printed, rinsed and post-cured, the object was sunk into a vial containing the resin (nBA: TcddA: DOT: ThermoTBD1: AIBN = 1:0.01:0.04:0.03:0.01). The vial was heated at 65°C for 24h, giving a stone-like object. Once this object is heated in an oven (150°C), the stone melt and revealed the statue. We thus washed it in THF to remove the degraded thermoset and then obtained the pure statue without any residual materials.

***Electric circuit***

An electric circuit was assembled using a DC regulated power supply (1.5 A, variable output voltage from 3 V to 12 V; EMC model) connected in series with a light-emitting diode (LED), a custom all-organic fuse embedded within a resistive heating coil, and a high-power resistive heating unit (Jeulin Evolution R24, 230 V, 1200 VA). The LED served as a visual indicator of current flow through the circuit. The temperature of the system was monitored and recorded using a Fluke 51 II digital thermometer.

**Figure S9.** Picture of the electric scheme used in the article

**References**

^1^ Bingham, N. M.; Roth, P. J. *Chemical Communications* 2019, 55, 55-58.

^2^ Shieh, P.; Zhang, W.; Husted, K. E. L.; Kristufek, S. L.; Xiong, B.; Lundberg, D. J.; Lem, J.; Veysset, D.; Sun, Y.; Nelson, K. A.; et al. *Nature* **2020**, 583, 542-547.

^3^ Zhang, S.; Xu, Z.; Husted, K. E. L.; Lundberg, D. J.; Brown, C. M.; Wang, Y.; Shieh, P.; Ko, K.; Moore, J. S.; Johnson, J. A. *Angew Chem Int Ed Engl* **2025**, 64, e202500104.
